# Dual Functionalization of Hyaluronan Dermal Fillers with Vitamin B3: Efficient Combination of Bio-Stimulation Properties with Hydrogel System Resilience Enhancement

**DOI:** 10.3390/gels10060361

**Published:** 2024-05-24

**Authors:** Alexandre Porcello, Michèle Chemali, Cíntia Marques, Corinne Scaletta, Kelly Lourenço, Philippe Abdel-Sayed, Wassim Raffoul, Nathalie Hirt-Burri, Lee Ann Applegate, Alexis Laurent

**Affiliations:** 1 Development Department, LOUNA REGENERATIVE SA, CH-1207 Geneva, Switzerland; c.marques@louna-aesthetics.com (C.M.); k.lourenco@louna-aesthetics.com (K.L.); 2 Plastic and Reconstructive Surgery, Ensemble Hospitalier de la Côte, CH-1110 Morges, Switzerland; michele.chemali@ehc.vd.ch (M.C.); wassim.raffoul@ehc.vd.ch (W.R.); 3 Regenerative Therapy Unit, Lausanne University Hospital, University of Lausanne, CH-1066 Epalinges, Switzerland; corinne.scaletta@chuv.ch (C.S.); philippe.abdel-sayed@chuv.ch (P.A.-S.); nathalie.burri@chuv.ch (N.H.-B.); lee.laurent-applegate@chuv.ch (L.A.A.); 4 STI School of Engineering, Federal Polytechnical School of Lausanne, CH-1015 Lausanne, Switzerland; 5 Center for Applied Biotechnology and Molecular Medicine, University of Zurich, CH-8057 Zurich, Switzerland; 6 Oxford OSCAR Suzhou Center, Oxford University, Suzhou 215123, China; 7 Manufacturing Department, LAM Biotechnologies SA, CH-1066 Epalinges, Switzerland; 8 Manufacturing Department, TEC-PHARMA SA, CH-1038 Bercher, Switzerland

**Keywords:** bio-stimulation, cohesivity attributes, cross-linked dermal fillers, dermal fibroblasts, functional characterization, hyaluronic acid, hydrogel system, niacinamide, skin collagen, viscoelasticity

## Abstract

Hyaluronic acid (HA) hydrogels are commonly used for facial dermal filling and for alternative medical aesthetic purposes. High diversity exists in commercial formulations, notably for the optimization of finished product stability, functionality, and performance. Polyvalent ingredients such as calcium hydroxylapatite (CaHA) or vitamin B3 (niacinamide) are notably used as bio-stimulants to improve skin quality attributes at the administration site. The aim of the present study was to perform multi-parametric characterization of two novel cross-linked dermal filler formulas (HAR-1 “Instant Refine” and HAR-3 “Maxi Lift”) for elucidation of the various functional impacts of vitamin B3 incorporation. Therefore, the HAR products were firstly comparatively characterized in terms of in vitro rheology, cohesivity, injectability, and resistance to chemical or enzymatic degradation (exposition to H_2_O_2_, AAPH, hyaluronidases, or xanthine oxidase). Then, the HAR products were assessed for cytocompatibility and in vitro bio-stimulation attributes in a primary dermal fibroblast model. The results showed enhanced resilience of the cohesive HAR hydrogels as compared to JUVÉDERM^®^ VOLBELLA^®^ and VOLUMA^®^ reference products in a controlled degradation assay panel. Furthermore, significant induction of total collagen synthesis in primary dermal fibroblast cultures was recorded for HAR-1 and HAR-3, denoting intrinsic bio-stimulatory effects comparable or superior to those of the Radiesse^®^ and Sculptra^™^ reference products. Original results of high translational relevance were generated herein using robust and orthogonal experimental methodologies (hydrogel degradation, functional benchmarking) and study designs. Overall, the reported results confirmed the dual functionalization role of vitamin B3 in cross-linked HA dermal fillers, with a significant enhancement of hydrogel system stability attributes and the deployment of potent bio-stimulatory capacities.

## 1. Introduction

Hyaluronic acid (HA) is a natural glycosaminoglycan and an important component of the extracellular matrix (ECM) of many tissues and organs such as the skin, limb joints, and eyes [1]. The disaccharide chemical structure of HA was described in 1954 by Meyer and Weissmann [2]. Many years after its discovery, HA continues to attract a great deal of interest in various fields. This prominence is mainly due to the well-documented safety, biocompatibility, and biodegradability of HA. Of note, this polymer is used in rheumatology, dermatology, oncology, ophthalmology, tissue engineering, aesthetic surgery, and cosmetology [2,3,4]. Specifically, HA-based dermal fillers may be considered as components of aesthetic and/or regenerative medicine formulations.

Dermal fillers, also known as injectable implants, soft tissue fillers, or wrinkle fillers, are defined by the US Food and Drug Administration (FDA) as medical device (MD) implants. They are notably approved for human use in enhancing facial aesthetics by creating a smoother and/or fuller appearance in the face [5]. Therein, most commercial HA-based products are indicated for facial volume loss due to age-related bone and fat resorption. Importantly, such devices are often obtained via HA chemical cross-linking with 1,4-butanediol diglycidylether (BDDE), as described in various manufacturing technologies (e.g., VYCROSS^®^, CPM^®^, Preserved Network^®^, IPN-Like^®^, or OxiFree™) [6,7,8,9,10,11,12,13]. The numerous variants for chemical modifications of HA, exploited in order to obtain physico-chemical advantages, are well-described in the literature [14]. Cross-linked HA dermal fillers are classified as «volumetric» or «volumizing fillers», which are temporary and biodegradable, and which act mechanically by direct and indirect tissular displacement [13,15].

Stimulatory fillers or bio-stimulators are often presented as skin collagen enhancers (i.e., products stimulating the synthesis of endogenous collagens by the body) and are classified as synthetic fillers. Two major commercial bio-stimulators are calcium hydroxylapatite (CaHA; e.g., Radiesse^®^) and poly-L-lactic acid (PLLA; e.g., Sculptra™). Of note, injectable PLLA has been used as a cosmetic filler since 1999, while CaHA was approved by the FDA in 2006 [16,17]. Clinically, CaHA is biodegradable and may be considered as a synthetic and long-lasting/semi-permanent dermal filler [18]. Similarly, Sculptra™ (i.e., PLLA microparticles) is used as a facial volumizer which promotes the gradual physiological deposition of fibrous tissues or collagen in the skin [16,19].

In the field of HA-based product characterization, a substantial body of literature compares the rheological properties of volumizing fillers [20,21,22,23]. Additionally, numerous studies evaluated and benchmarked various finished product bio-physical attributes (e.g., extrusion forces, cohesivity, and swelling ratio). Importantly, the hydrogel characterization methodologies were largely consistent across studies, with minimal variations in approaches and results [22,23,24,25,26,27,28,29,30,31,32]. However, experimental unraveling of the degradation mechanisms in cross-linked HA-based fillers is highly complex and challenging. Specifically, methodological variations (i.e., polymer breakdown model and analytical means) were reported for characterizing such HA-based system degradation processes [22,23,24,25,26,27,28,29,30,31,32].

HA hydrogel physiological degradation is mainly mediated by specific catabolic enzymes (i.e., hyaluronidases, HYAL) or by reactive oxygen species (ROS) and secondly by mechanical stress or thermal hydrolysis [13,32,33,34,35]. Notably, the extent of polymer degradation induced by HYAL is influenced by multiple factors. These include the retained concentration of the enzyme (e.g., from 5 to 100 U/mL), the specific type of hyaluronidase (e.g., ovine, bovine, or commercial products including Hyalase^®^), the duration of exposure, and the methods for quantification of the degradation (e.g., time-lapse microscopy, in vivo assay, colorimetric assay, or rheology) [13,21,25,31,36,37,38].

Furthermore, when examining oxidative stress-induced HA-based hydrogel degradation, the diversity in the applied experimental methodologies is high. Therein, H_2_O_2_ alone or in combination with copper (II) or iron (II) is predominantly used for inducing ROS-mediated degradation. In these studies, rheological values are often used as the primary outcome measure to assess the extent of system degradation [13,25,39,40,41]. The selection of H_2_O_2_ as an oxidant source stems from its role as a physiological ROS within the skin. Notwithstanding, alternative oxidant sources exist, such as xanthine oxidase/xanthine, nitric oxide (NO), or 2,2’-azobis(2-amidinopropane) dihydrochloride (AAPH), which are small-molecule ROS generators [34,39,42,43,44]. The oxidative stress generated by ROS in the skin can cause premature aging and lead to skin damage (e.g., hyperpigmentation or dryness), hence the success of antioxidants in the cosmetic industry [44,45,46]. As concerns bio-stimulators, the methods of functional characterization are different and scarcer than for HA-based dermal fillers. Nevertheless, various clinical and histological reports have shown the effects of Radiesse^®^ and Sculptra^™^ [16,47,48,49,50]. However, in vitro studies aiming to quantify the effects of Radiesse^®^ and Sculptra^™^ on collagen synthesis by dermal fibroblasts are scarcely reported [16].

The present study was designed during the development of a novel dermal filler technology (i.e., “Hyaluronic Acid-Reticulated” [HAR] boosting fillers) based on BDDE-cross-linked HA and vitamin B3 (niacinamide). Specifically, the HAR formulas were tailored to combine the volumizing attributes of cross-linked HA and the bio-stimulatory properties of vitamin B3. Additionally, the latter is an antioxidant which may exert a protective effect against the degradation of HA polymer chains [51,52,53]. Thus, the aim of this study was to perform multi-parametric in vitro characterization of the HAR-1 “Instant Refine” and HAR-3 “Maxi Lift” dermal fillers and to provide comparative functional results (system stability, bio-stimulatory attributes) of high translational relevance. A primary hypothesis of the study was that vitamin B3 incorporation in cross-linked HA systems results in a dual enhancement of hydrogel stability and in vitro bioactivity. A secondary hypothesis of the study was that the use of orthogonal methods for oxidative degradation assessment provides an enhanced understanding of in situ hydrogel catabolism in the skin.

Generally, the originality and significance of the study resided in the combination of the described methodological approaches for commercial HA- and vitamin B3-based dermal filler characterization. In detail, the reported characterization of the HAR formulations included assays which are typically used for traditional dermal fillers as well as those used for bio-stimulator products. To the best of our knowledge, we are the first to analyze functionalized dermal filler degradation by rheology using three ROS sources, as other groups usually only use H_2_O_2_. Therefore, the study novelly reported several method combinations and complementary in vitro datasets for an enhanced understanding of the in situ fate and effects of the investigated devices. Furthermore, this study was the first to report experimental data on cross-linked dermal filler hydrogels containing vitamin B3, manufactured for the first time with the Boost and Fusion technology (i.e., HAR hydrogels). Overall, the present study contributed to advancing and standardizing the methods for polyvalent hydrogel function-oriented development, ultimately aiming to bring safe and performant products to patients.

## 2. Results and Discussion

### 2.1. HAR Hydrogel Systems: Technical Benchmarking Study and Product Formulation Analyses

Prior to in vitro laboratory testing, the HAR hydrogels were firstly technically benchmarked and analyzed from a product formulation viewpoint. Importantly, when developing a novel HA-based hydrogel technology, the design considerations always depend on the intended clinical use(s) [25]. Furthermore, in the field of aesthetic medicine, product design considerations are critically related to the choice of raw materials (i.e., HA source, cross-linking agent, additives, and anesthetics). Specifically, the starting HA polymer concentration played a major role in the first steps of HAR hydrogel development, along with the time and temperature of the BDDE cross-linking reaction (i.e., performed at basic pH). Of note, the HA starting material for both of the HAR formulations had an intrinsic viscosity of 2.5 m^3^/kg (i.e., determined following Ph. Eur., in a pH 7.0 phosphate buffer). As regards the clinical indications, HAR-1 was mainly designed to be injected in the dermis, while HAR-3 was designed for subcutaneous fat tissue or for supraperiostic zone administration (Table 1).

From a detailed formulation and manufacturing process standpoint, both HAR hydrogels were obtained using the Boost and Fusion technology (Table 2).

The modern landscape of HA-based dermal fillers is commercially populated by multiple injectable products [25]. Therein, the JUVÉDERM^®^ brand (i.e., VYCROSS^®^ technology) is often cited as a gold standard [6]. Notably, JUVÉDERM^®^ VOLUMA^®^ with lidocaine is intended to be used to restore volume in the face [6]. On the other hand, JUVÉDERM^®^ VOLBELLA^®^ with lidocaine is intended for enhancement and pouting of the lips (i.e., to correct volume loss via labial mucosal injection), to treat perioral lines and oral commissures, and to correct infraorbital skin depressions [6]. Importantly, both JUVÉDERM^®^ products are cross-linked with BDDE (Table 1). Due to respective technical similarities between the VOLBELLA^®^ and HAR-1 hydrogels or between the VOLUMA^®^ and HAR-3 hydrogels, the JUVÉDERM^®^ products were retained for analysis as reference commercial comparators (Table 1). Furthermore, the JUVÉDERM^®^ products were retained for analysis as they do not contain vitamin B3 or any other antioxidant excipient, thereby enabling to individually/specifically assess the functional impacts of vitamin B3 incorporation in the HAR systems. Overall, this choice of commercial comparators was made to enhance the translational relevance of the study and to experimentally investigate the primary hypothesis of the study.

### 2.2. HAR Hydrogel Rheological Characterization

**Figure 1:**
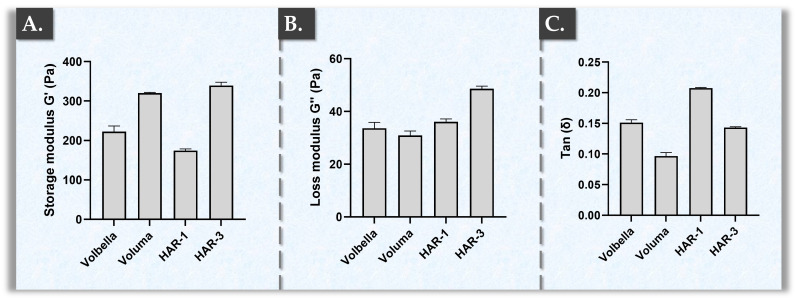
Results of basic rheological characterization studies for the hydrogels of interest. (**A**) Storage modulus G′ values for the undiluted samples at 25 °C. (**B**) Loss modulus G″ values for the undiluted samples at 25 °C. (**C**) Tan delta (tan δ) values for the undiluted samples at 25 °C. Measurements were performed in triplicate and standard deviations were reported as error bars around mean values. Detailed results of the statistical analyses are presented in Table S1. Pa, Pascals.

In order to firstly perform basic physico-chemical characterization of the HAR-1 and HAR-3 hydrogels, oscillatory rheology analyses were performed. The results enabled to experimentally compare the storage modulus (G′), loss modulus (G″), and tan δ values of the two HAR systems to those of the VOLBELLA^®^ and VOLUMA^®^ controls (Figure 1).

Among the tested hydrogel products, HAR-3 and VOLUMA^®^ exhibited the highest storage modulus G′ values (Figure 1A, Figure S1). The values of the VOLUMA^®^ samples were linked to a relatively high reported percentage of cross-linking (i.e., a larger quantity of incorporated BDDE) in the VOLUMA^®^ hydrogel [29,54]. Specifically, the VYCROSS^®^ technology is known to leverage important polymer cross-linking percentages as compared to alternative commercially available products [29,54]. On a technical note, the degree of cross-linking in JUVÉDERM^®^ products refers to how many covalent bonds are established between two HA molecules on an average basis [54]. The rheological values of HAR-3 were linked to cross-linking specificities and the possible interactions between the HA chains and vitamin B3 (Figure S2). As G′ corresponds to the contribution of the resistance to deformation of HA polymer chains (i.e., the gel firmness in dynamic conditions), it may be expected that higher cross-linking degrees in the hydrogel would lead to higher G′ values [20,24]. Parallelly, the G″ modulus corresponds to the contribution of the liquid phase of the system and represents the inability of the hydrogel to recover its shape completely after shear deformation [55].

Regarding the experimental loss moduli G″ values, the VOLUMA^®^, VOLBELLA^®^, and HAR-1 samples were found to be in the same range of 31–37 Pa (Figure 1B). Contrastingly, HAR-3 exhibited significantly higher loss moduli G″ values. Notably, the quantitative rheological attributes which were determined for the VOLBELLA^®^ and VOLUMA^®^ products were found to be consistent with the literature [55].

When focusing on HAR-1, the storage modulus results showed lower values than for the other products (Figure 1). Namely, HAR-1 presented an average G′ value of 174 Pa, which was approximately 1.9 times lower than that of HAR-3 and 1.3 times lower than that of VOLBELLA^®^ (Figure 1A). Interestingly, HAR-1 displayed significantly higher tan δ values (Figure 1C). HAR-3 and VOLBELLA^®^ were in a similar range and VOLUMA^®^ was the only product presenting significantly lower tan δ values (i.e., averaging at 0.096; Figure 1C). Of note, the G″/G′ ratio yields the tan δ value of the system, which inversely correlates to the elasticity of the HA filler. Importantly, tan δ is a good indicator for determining whether the filler can be injected more superficially (i.e., higher tan δ values) or deeper (i.e., lower tan δ values) in/under the skin [55].

In general, higher degrees of polymer cross-linking render the gel “harder”, augment the persistence of the filler after injection into soft tissues, and lower the hydrophilicity of the filler. Notwithstanding, manufacturers are not required to disclose the applied degree of cross-linking for a given product. Furthermore, there are various cross-linking degree measurement methods (e.g., size-exclusion chromatography with mass spectrometry [SEC-MS] or nuclear magnetic resonance [NMR]), wherein a lack of methodological homogeneity exists. Currently, there is no consensus on the optimal method to use in order to determine these parameters [12,55,56].

For a linear HA-based hydrogel at 10 mg/mL or 20 mg/mL HA, the addition of vitamin B3 at a concentration of 10 mg/mL does not alter its rheology. Similar observations were previously reported for a solution of hydroxypropyl methylcellulose at various concentrations of vitamin B3 [57]. Thus, it may be considered that vitamin B3 (i.e., as an additive at a concentration of 7.5 mg/mL) does not play a significant role in the rheology of the system. However, in the manufacturing process of the HAR-1 and HAR-3 gels, vitamin B3 is added in two stages, namely during the cross-linking phase (i.e., at basic pH) and after the cross-linking phase (i.e., at neutral pH). Therefore, it is difficult to compare this process to that of a simple excipient which is added by terminal incorporation. Of note, vitamin B3 more likely acts as a salt, with possible hydrogen bonding (i.e., the nitrogen atom on the pyridine ring and the carbonyl oxygen atom on the amide group) with the hydrophilic groups of the HA polymer (Figure S2) [57,58,59].

Of note, the rheological values of commercial products are generally highlighted by manufacturers as functional proxies. From a rheological point-of-view, the HAR-1 hydrogel can also be compared to the STYLAGE^®^ M product from Vivacy (Paris, France). Specifically, STYLAGE^®^ M is composed of 20 mg/mL HA and presents G′ values of 195 Pa and G″ values of 35 Pa at 1 Hz. In contrast, the HAR-3 product can also be compared to the Teosyal RHA^®^ 4 product from Teoxane (Geneva, Switzerland) or to the STYLAGE^®^ XL product from Vivacy [60,61]. Specifically, RHA^®^ 4 is composed of 23 mg/mL HA, and displays mean G′ values of 346 Pa and G″ values of 62 Pa at 5 Hz, whereas STYLAGE^®^ XL is composed of 26 mg/mL HA and presents G′ values of 290 Pa and G″ values of 46 Pa at 1 Hz [60,61]. Overall, the gathered rheological data confirmed that both HAR hydrogels were comparable in terms of physical behavior with widely applied commercial dermal fillers (Figure 1).

### 2.3. HAR Hydrogel Multi-Parametric Accelerated Degradation Assays

**Figure 2:**
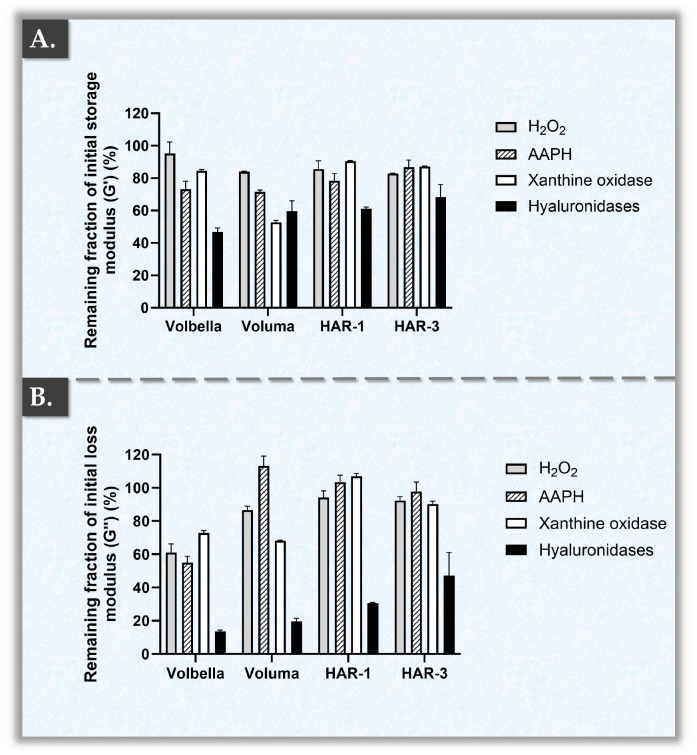
Results of accelerated hydrogel degradation studies expressed as endpoint residual fractions of the rheological attributes of the samples as compared to their initial (i.e., unchallenged) values. (**A**) Experimental endpoint storage modulus G′ values for the challenged samples. (**B**) Experimental endpoint loss modulus G″ values for the challenged samples. All the samples were parallelly exposed to strong oxidant sources and to hyaluronidases. After 10 min of exposure, the samples were analyzed in oscillatory rheology at 22 °C with a frequency of 1 Hz. Measurements were performed in triplicate and standard deviations were reported as error bars around mean values. The corresponding complex viscosity η* values are presented in Figure S3. Detailed results of the statistical analyses are presented in Tables S2–S4.

In vivo, HA hydrogel degradation is mainly mediated by HYAL and ROS. In order to capture a more advanced and thorough understanding of the biophysical attributes of the HAR hydrogels, accelerated product degradation assays were performed. These studies were specifically designed to include standardized orthogonal methods (i.e., oxidant- and enzyme-mediated polymeric system degradation) and to provide quantitative data of enhanced translational relevance. Specifically, while HYAL- or H_2_O_2_-based in vitro hydrogel challenge assays are of use for basic product benchmarking, such experimental designs fail to comprehensively mimic or approximate the multi-parametric catabolic drivers at play in vivo [21,26]. The HAR-1, HAR-3, and the two JUVÉDERM^®^ control products were therefore parallelly submitted to simulated in vitro challenge using H_2_O_2_, AAPH, hyaluronidases, or xanthine oxidase sources. Interestingly, distinctive endpoint rheological behaviors were evidenced for the respective hydrogel systems, depending on the specific challenge item (Figure 2).

As previously mentioned, two major mechanisms that contribute to the degradation of HA-based gels are oxidative reactions and enzymatic catabolism (i.e., primarily via hyaluronidases). Specifically, the degradation of HA-based gels is a result of the cleavage of hyaluronic acid chains, leading to a decrease in the rheological attribute values (e.g., G′ and G″ moduli) of the system [13,35,39,40,41]. Firstly, when considering the reduction in storage moduli G′ associated with exposition to H_2_O_2_, the compared products did not behave significantly differently (Figure 2A). Contrastingly, the remaining fractions of the loss moduli G″ were found to be significantly lower for the VOLBELLA^®^ product as compared to the other tested products (Figure 2B, Table S3). Of note, H_2_O_2_ is the most common and simplest ROS generator to be used when evaluating the in vitro oxidative degradation of HA [25].

Secondly, when comparing the reductions in storage moduli G′ induced by AAPH (i.e., a free radical generator) exposure, HAR-3 surprisingly exhibited significantly higher remaining fractions as compared to VOLUMA^®^, VOLBELLA^®^, and HAR-1 (Figure 2A, Table S2). Furthermore, VOLUMA^®^ was recorded as the most sensitive formulation in terms of storage modulus G′ under the AAPH challenge (Figure 2A). Then, when analyzing the remaining fractions of loss moduli G″, VOLBELLA^®^ exhibited significantly lower viscosity values as compared to HAR-1, HAR-3, and VOLUMA^®^ products (Figure 2B, Table S3). Surprisingly, the ratio of G″ to G′ (i.e., tan δ values, elasticity proxy of the material) of the VOLUMA^®^ product increased by 63% after exposure to AAPH. Such experimental tan δ values were the highest for the VOLUMA^®^ samples.

Thirdly, when comparing the extent of the oxidative degradation induced by the mixture of xanthine/xanthine oxidase, HAR-1 and HAR-3 displayed significantly higher remaining fractions of G′ and G″ (Figure 2, Tables S2 and S3). These results indicated superior resilience of the HAR hydrogels against xanthine oxidase as compared to VOLUMA^®^ (i.e., close to 1.7 times for G′ and close to 1.3 times for G″, Figure 2, Tables S2 and S3). Interestingly, VOLBELLA^®^ displayed similar extents of degradation to those of both HAR formulations for G′ but similar degradation to that of VOLUMA^®^ for G″ (Figure 2). Overall, the presented results confirmed oxidant-specific catabolic behaviors of the investigated cross-linked HA hydrogels (Figure 2). Therefore, the use of several oxidative challenge items is highly recommended in accelerated stability studies or in simulations of in vivo product degradation to comprehensively characterize the resilience of a given system.

In vivo, free radicals are generated as radicals of lipids, proteins, and DNA alongside various ROS, which are also categorized as free radicals (e.g., superoxide anion [O_2_•^−^], hydroxyl radical [•OH], nitric oxide radical [•NO], and peroxynitrite anion [ONOO^−^]). Oxidative stress is characterized by an elevated level of ROS generation and the presence of other oxidative species, which exceed the capacity of the cellular antioxidant mechanisms to neutralize them [44]. Xanthine oxidase is an enzyme that catalyzes the production of the superoxide anion (O_2_•−) and which is expressed in skin cells [44,62,63]. AAPH is a water-soluble compound primarily used as a source of peroxyl radicals (ROO•). It is commonly employed to induce oxidative stress and cellular senescence in skin cells or as an oxidation model in various studies [64,65,66]. Hydrogel product rheological attributes, particularly the storage modulus G′, influence the in vivo performance of dermal fillers. Namely, rheology provides a straightforward and rapid method to assess a product’s resistance to oxidative stress by using the parameters G′ and G″ and an appropriate oxidant source.

Fourthly, a few significant differences were evidenced between the groups in residual endpoint storage modulus G′ values following HYAL exposure (Figure 2A, Table S2). Therein, HAR-3 displayed higher remaining fractions of storage modulus G′ as compared to the other tested products (Figure 2A). Of note, VOLBELLA^®^ displayed the most extensive enzymatic degradation, as it was the only product with a remaining average fraction of storage modulus G′ below 50% (Figure 2A). Interestingly, more pronounced differences were recorded between the groups in terms of residual loss modulus G″ (Figure 2B, Table S3). Therein, HAR-3 exhibited higher resistance towards HYAL, and VOLUMA^®^ showed lower resistance (Figure 2B). Notwithstanding the reported results on product resilience toward enzymatic degradation, it is underscored that the performance and longevity of HA-based fillers are more closely associated with the storage modulus G′ (Figure 2) [24,25].

Interestingly, HAR-1 and HAR-3 exhibited similar remaining rheological fraction values in oxidative environments (i.e., low dispersion of values between assay types for given products) despite their differences in formulation (Figure 2, Table 1 and Table 2). Namely, both HAR products displayed remaining fractions > 78% for both moduli following exposure to three different ROS sources (Figure 2). In contrast, VOLBELLA^®^ displayed a disparity in sensitivity to the various ROS sources, with values dropping as low as approximately 52% (Figure 2). Generally, it was set forth that the HAR hydrogels presented enhanced global resilience towards oxidant-mediated degradation as compared to the JUVÉDERM^®^ control products.

Vitamin B3 is known for its antioxidant properties [52,53,67]. However, the presence of an antioxidant molecule does not necessarily equate to the effective protection of HA-based gels from degradation under oxidative stress. For instance, vitamin C is recognized for its potent antioxidant capacity but can significantly degrade HA [68]. Of note, the precise mechanism of interaction between vitamin B3 and HA polymers under high concentrations of H_2_O_2_, AAPH, or xanthine oxidase is not yet fully understood. This mechanism may be influenced by hydrogen bonding and a limiting effect on HA chain breakdown by vitamin B3.

Overall, the gathered experimental results confirmed that cross-linked HA hydrogel formulations containing vitamin B3 benefit from a protective effect against oxidative stress but not against the action of hyaluronidases (Figure 2). Such datasets confirmed the technical rationale of incorporating vitamin B3 in the HAR formulations to benefit from its polyvalent antioxidant attributes and effects. Namely, vitamin B3 may be considered as a product stability and performance enhancer due to its ancillary internal action on the cross-linked hydrogel system. From a methodological viewpoint, the use of close commercial comparators (i.e., the JUVÉDERM^®^ control products, technically similar to the HAR systems but without vitamin B3) enabled us to specifically experimentally study the functional impacts of vitamin B3 incorporation. However, further research is warranted, notably around the exact mechanisms of chemical interaction and incorporation of vitamin B3 in the HA polymer network (Figure S2). The unraveling of such mechanisms could potentially shed more light on the overall functional contributions of vitamin B3 in HA-based hydrogels.

Finally, this was the first study to use advanced rheological analysis to assess dermal filler degradation by multiple ROS, offering new insights into the stability and resilience of HA-based dermal fillers under complex oxidative stress. Therefore, this contribution addressed significant gaps in the understanding of dermal filler degradation and introduced innovative methodologies to the field.

### 2.4. HAR Hydrogel System Cohesivity and Injectability Assays

In order to further functionally characterize the hydrogel systems of interest, cohesivity quantifications were performed. This biophysical attribute characterizes how a dermal filler behaves in the form of a gel deposit once it is injected and subjected to mechanical forces and constraints in the body. Of note, when high compression is applied to a low-cohesivity gel, there is a risk of detachment/separation from the original deposit location, which can potentially result in adverse dermal filler migration. Contrastingly, when high compression is applied to a high-cohesivity gel, the deposit resists displacement more easily and retains its original shape [25,27].

**Figure 3:**
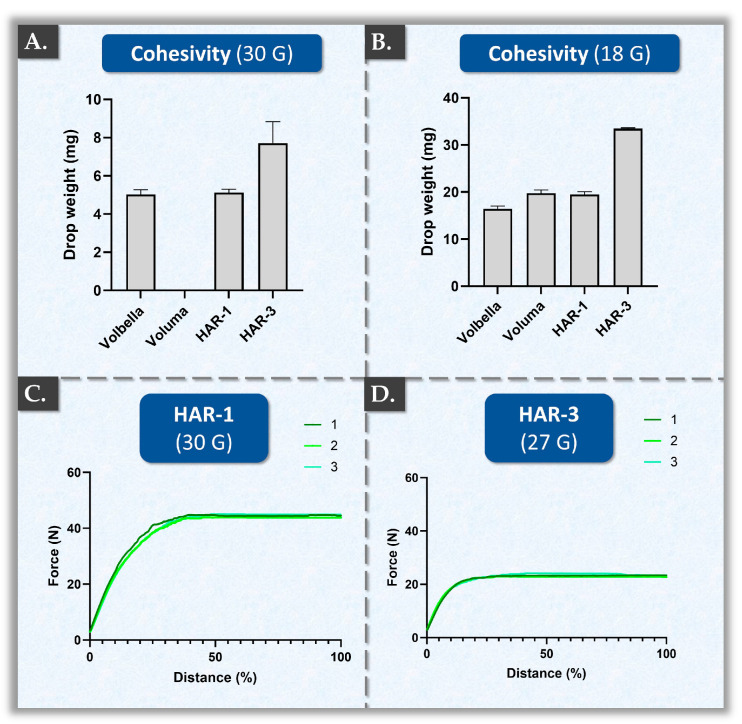
Comparative hydrogel system biophysical characterization results. (**A**) System cohesivity determined by the drop-weight method (30 G needle, 12 mm·min^−1^ extrusion). An illustration of the investigated hydrogels is presented in Figure S4. An illustration of the experimental setup is presented in Video S1 (i.e., VOLUMA^®^ product). (**B**) System cohesivity determined by the drop-weight method (18 G needle, 7.5 mm·min^−1^ extrusion). Detailed results of the statistical analyses are presented in Table S5. (**C**) Injection force profiles of HAR-1 in an automated measurement setup. (**D**) Injection force profiles of HAR-3 in an automated measurement setup. Injectability results were presented for three distinct product syringes, corresponding to profiles N°1, N°2, and N°3. Injectability assays were performed at ambient temperature using a constant plunger rod actuation speed of 1 mm·s^−1^. All measurements were performed in triplicate. G, gauge.

From a dermal filler formulation standpoint, system cohesivity is a function of both the HA concentration and the degree of polymer cross-linking. Specifically, using the same cross-linking technology, the action of increasing either the HA concentration or the HA cross-linking degree generally leads to increases in system cohesivity. For the experimental assay, the drop-weight method was retained, as there was a good correlation with the perceived cohesion attributes of the hydrogels (Figure 3) [27].

Importantly, drop-weight cohesivity values are dependent upon hydrogel extrusion speed and the dimensions of the needle. Quantitatively, with a 30 G needle and a constant plunger speed of 12 mm·min^−1^, the mean drop-weight values of HAR-1 and VOLBELLA^®^ were almost identical (Figure 3A). In the case of HAR-3, however, mean values 1.5 times higher than those of VOLBELLA^®^ and HAR-1 were recorded (Figure 3A). Of note, no experimental values were obtained for the VOLUMA^®^ product in this setup because it did not form drops but a continuous filament (Video S1). A possible technical explanation for this observation is the presence of a linear HA phase in the VOLUMA^®^ hydrogel system. Specifically, adding and mixing a linear HA phase to a cross-linked product notably facilitates its injectability. However, no detailed quantitative aspects of this linear HA phase are available, as manufacturers do not generally disclose HA molecular weight ranges, degrees of cross-linking, or linear HA phase contents.

Quantitatively, with an 18 G needle and a constant plunger speed of 7.5 mm·min^−1^, the mean drop-weight values of HAR-1 and VOLUMA^®^ were almost identical (Figure 3B). Therein, the cohesivity values of VOLBELLA^®^ were significantly lower than those of HAR-1 (i.e., 16.6 mg per drop for VOLBELLA^®^ versus 19.5 mg per drop for HAR-1, Figure 3B, Table S5). Of note, HAR-3 presented a significantly higher average drop-weight as compared to the other products, confirming that the HAR-3 product is characterized by superior cohesiveness (Figure 3A,B, Table S5). Importantly, the use of 18 G needles and an extrusion speed of 7.5 mm·min^−1^ are standard parameters that are frequently reported in the literature. The experimental values obtained for VOLUMA^®^ and VOLBELLA^®^ were almost identical to those previously published [27]. Interestingly, as compared to VOLUMA^®^, HAR-1 contains a lower hyaluronic acid concentration, presents lower G′ values, uses less BDDE and a lower degree of modification, but exhibits higher cohesiveness values (Figure 3, Table 1 and Table 2). These results confirmed the potential role of vitamin B3 as a potent stabilizer of the HA matrix, which acts by enhancing the internal adhesion forces that hold together the individual cross-linked HA units composing the system via hydrogen bonds. Overall, the HAR-1 and HAR-3 formulations allow for a reduction in the amount of incorporated BDDE while maintaining high cohesivity properties.

Regarding the injectability of the HAR hydrogels, both products showed high homogeneity in their injection force profiles at a relatively high speed of injection (Figure 3C,D). The injection force profile of an empty syringe (i.e., without any product) served as a control, and a representative dataset is presented in Figure S5. For optimal data comparability, the experimental setup of this test was identical to that of a previous study by the authors [69]. Of note, HAR-1 demonstrated significantly higher injection forces as compared to HAR-3 (Figure 3C,D, Table 3).

This difference was notably attributed to HAR-1 being administered using a 27 G needle, whereas HAR-3 was injected using a 30 G needle (i.e., 1.3 times narrower than 27 G needles). Nevertheless, in comparison with previously reported injection force profiles, those of HAR-1 and HAR-3 did not show any plateau heterogeneity (i.e., achievement of seamless injectability), wherein the three experimental replicates overlapped (Figure 3C,D) [69].

Interestingly, in terms of average plateau injection force, the HAR-3 product fell within the same range as the VOLBELLA^®^ product (i.e., 20–25 N) and the HAR-1 product fell within the same range as the VOLUMA^®^ product (i.e., 40–50 N; Figure 3C,D) [69]. Of note, the plateau injection force does not linearly correlate with the HA concentration or the needle gauge, suggesting that the product extrusion force is likely determined by a complex interplay of factors [69]. Such factors include the HA concentration, needle gauge, formulation parameters, degree of cross-linking, percentage of HA linear phase, and the specific technology used during product manufacturing. Importantly, hydrogel injectability is an important functional attribute of dermal fillers [69]. Indeed, various issues may occur in cases of non-homogeneity during the viscous regime (i.e., linear injection phase) of the injection process. Specifically, hydrogel system non-homogeneity can lead to an uneven in situ distribution of the product, potentially resulting in undesirable events (e.g., Tyndall effect) and uneven aesthetic outcomes [29].

### 2.5. HAR Hydrogel Bio-Stimulatory Attribute Assessment in a Cutaneous Cell Model

The first part of the study enabled us to comparatively characterize several hydrogel intrinsic attributes or the internal effects of the incorporated excipients (i.e., wide-spectrum protective antioxidant action of vitamin B3; Figure 1, Figure 2 and Figure 3). Then, the second part of the study was designed to assess the external effects or the interactions of the investigated products with relevant biological entities. Therefore, based on the clinical indications of the HAR-1 and HAR-3 products, an in vitro primary dermal fibroblast model was established. Similarly to the first part of the study, the cell-based assays were designed around the most relevant controls and commercial reference products available. Therefore, in the context of bio-stimulatory effect investigation, gold-standard commercial bio-stimulants such as Radiesse^®^ and Sculptra™ were included for analysis (Table 4).

Specifically, the Radiesse^®^ and Sculptra™ comparator products were retained because they have been clinically used for several years, have displayed clinical efficacy results, and were historically assessed as being safe for human use. Based on professional market reviews, Radiesse^®^ and Sculptra™ were determined to be the best available options for bio-stimulant treatments on the market, which is why they were included as positive controls in the study. Overall, this choice of commercial comparators contributed to enhancing the translational relevance of the study and was preferred over the use of PLLA or CaHA chemical reference standards, for example.

**Figure 4:**
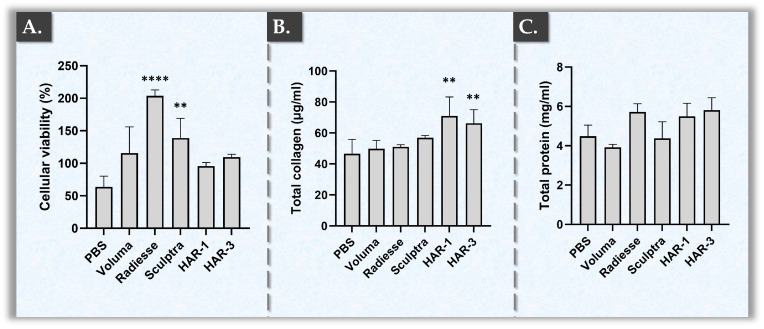
Comparative product bio-stimulatory effect assessments in an in vitro cell-based model. (**A**) Cellular viability of primary dermal fibroblasts incubated in contact with the samples for 96 h. (**B**) Absolute values of the total collagen produced by primary dermal fibroblasts incubated with the samples for 96 h. (**C**) Absolute values of the total proteins produced by primary dermal fibroblasts incubated with the samples for 96 h. Measurements were performed four times and standard deviations were reported as error bars around mean values. Significant differences with the PBS control groups were evidenced by asterisks (i.e., two asterisks “**” corresponded to a *p*-value between 0.001 and 0.01, four asterisks “****” corresponded to a *p*-value inferior to 0.0001). Photographic and mechanistic illustrations of the experimental setup are presented in Figures S6–S8. Detailed results of the statistical analyses are presented in Tables S6–S8. PBS, phosphate-buffered saline.

Firstly, the cellular viability of primary dermal fibroblasts that were directly exposed to the investigated products was assessed in a WST-1 mitochondrial activity assay. Therein, all of the included product groups retained viability after 96 h of incubation, with only the PBS control showing a decrease in average cell viability (i.e., 63.5%; Figure 4A).

Interestingly, the two reference products, which are classified as bio-stimulators (i.e., Radiesse^®^ and Sculptra™), demonstrated higher cellular viability values than those based on HA (i.e., HAR-1, HAR-3, and VOLUMA^®^; Figure 4A). Specifically, Radiesse^®^ showed the highest cellular metabolic activity levels, followed by Sculptra™ (Figure 4A). Contrastingly, VOLUMA^®^, HAR-1, and HAR-3 were in the same range and did not show significant differences among them (Figure 4A, Table S6).

Generally, the observed results of cellular viability/metabolic activity could be explained by the differential formulation attributes of the investigated products (Figure 4A, Table 4). Specifically, the CaHA and PLLA particles, respectively, contained in Radiesse^®^ and Sculptra™, are known to stimulate cellular activity [16,17,18,19]. Furthermore, the results related to cross-linked HA were consistent with the literature [25]. However, it is important to note that the tests were conducted on large multi-well plates due to the difficulty of viscous product removal in smaller wells. Specifically, Radiesse^®^ was particularly difficult to eliminate, as it adhered to the bottom and sides of the assay well (Figure S6). Therefore, Radiesse^®^ syringes were diluted five-fold to avoid potential bias during the analyses, especially for colorimetric readouts (Table 4). Additionally, to ensure uniformity of endpoint analysis, all wells were rinsed six times with 0.5 mL of PBS before further processing.

As regards total proteins and collagen quantification, HAR-1 and HAR-3 stimulated their production more effectively than VOLUMA^®^ and PBS (Figure 4B,C). Such results could notably be attributed to the action of vitamin B3, which is well-known for stimulating collagen production, a keynote of skin repair and regeneration [52,53,67]. Furthermore, despite their differences in HA contents, no significant differences were observed in terms of total proteins and collagens between HAR-1 and HAR-3, underscoring the major role of vitamin B3 in collagen and total protein synthesis stimulation (Figure 4B,C, Table 1, Table S7 and S8). When compared to the reference bio-stimulators, HAR-1 and HAR-3 indicated a trend towards higher collagen concentrations, but no significant differences with Sculptra™ were found (Figure 4B, Table S7).

Generally, the dual functionalization of cross-linked HA-based hydrogels by vitamin B3 necessitated dual characterization, with respective standards or commercial comparators for both aspects (i.e., physical attributes and bio-stimulation effects). Therein, Radiesse^®^ and Sculptra™ were selected as comparators for the bio-stimulation assays due to their international and widespread clinical use as injectable bio-stimulators. Interestingly, HAR-1 and HAR-3 demonstrated significantly greater collagen production induction than Radiesse^®^ and showed a superior trend to Sculptra™ (Figure 4B, Table S7). In terms of total proteins, Radiesse^®^ exhibited comparative values within the same range as both HAR products, while Sculptra™ showed a trend of lower values (Figure 4C, Table S8). Importantly, despite significantly higher cellular activity levels for Radiesse^®^ as compared to the two HAR products, the collagen quantity was significantly greater for the latter (Figure 4A,B, Tables S6 and S7). Therefore, the reported results confirmed that both of the HAR products boosted fibroblasts to produce more collagen, yet they did not exert significant effects on cellular metabolic activity (i.e., a characteristic of classical bio-stimulants; Figure 4, Figure S7).

Of note, the tested primary dermal fibroblasts retained their characteristic morphology in vitro following incubation with the investigated products (Figure S8). Previously, vitamin B3/niacinamide was proven to prevent cellular senescence (i.e., modeled by a decrease in protein levels, including collagen/elastin) in fibroblast models [67,70]. The anti-senescence effects of niacinamide largely stem from its anti-inflammatory properties, which include reducing inflammatory mediators and inhibiting mast cell degranulation [67,71,72]. Additionally, as a precursor to NAD^+^, niacinamide helps to prevent the depletion of cellular energy and regulates the cell’s redox status, both of which are essential for collagen synthesis [53,67,73]. Overall, the gathered experimental results demonstrated that the vitamin B3 present in HAR-1 and HAR-3 conferred multifactorial benefits to the HAR hydrogel systems, notably in terms of potent oxidative resilience and pro-collagenesis (Figure 2 and Figure 4).

From a specific mechanistic viewpoint, the bio-stimulant actions of CaHA, PLLA, and vitamin B3 were well-described in the literature, among which the aforementioned “skin collagen enhancement” attributes. Notably, the resorbable PLLA of the Sculptra™ stimulatory filler was described to exert its effects by promoting neo-collagenesis upon injection [19]. The detailed mechanism of action is thought to comprise the induction of a foreign-body reaction characterized by increased macrophage, mast cell, and lymphocyte presence. In turn, this cellular response is set forth to slowly degrade the product, increase fibroblastic activity, and result in gradual neo-collagenesis [74]. By immunofluorescence staining, Stein et al. confirmed that PLLA-induced augmentation is most likely based on capsule formation orchestrated by macrophages, (myo-)fibroblasts, and collagen type I and III fibers [75].

As regards the CaHA microspheres in Radiesse^®^, in vitro analyses showed a significant increase in COLIII expression by dermal fibroblasts, which was 123% higher than baseline at 24 h post-incubation. Interestingly, this enhanced expression of COLIII was observed only in fibroblasts that were in direct contact with CaHA, indicating that the recorded increase in collagen production was rather due to the activation of more fibroblasts than to a change in the rate of cellular expression [76]. Furthermore, a systematic review by Amiri et al. compiled data on the skin regeneration mechanisms related to CaHA, focusing particularly on neo-collagenesis. The authors highlighted supporting evidence that demonstrated that collagen production increased in proportion to the amount of applied CaHA, further substantiating the potential of CaHA in skin regenerative applications [77].

Finally, concerning the activity of vitamin B3, we recently described that the intrinsic antioxidant activity of the molecule contributes to cellular homeostasis, thereby maintaining skin ECM integrity [67]. As mast cell degranulation leads to fibroblast senescence, vitamin B3 contributes to normal fibroblast activity maintenance by its stabilizing effects. Of note, vitamin B3 also inhibits MMPs due to its antioxidant and anti-inflammatory activities. Furthermore, it was shown that vitamin B3 inhibits elastase activity, preserving the integrity of elastin in cutaneous ECM [67]. Overall, while the reported results did not mechanistically describe the exact bio-stimulatory pathway of the considered HAR hydrogel systems, strong parallels could be made with previous reports on the beneficial effects of selected ingredients. Interestingly, the reported functional performance of the HAR systems in experimental benchmarking with PLLA- and CaHA-based products confirmed their significant intrinsic bioactivity in a relevant cutaneous cell-based model (Figure 4). Thus, the present study set forth several datasets confirming the multiple functionalization roles of vitamin B3 and supporting its use as a versatile, functional ingredient in medicalized skincare.

### 2.6. Study Limitations and Future Perspectives

As regards the main identified limitations of the present study, it was outlined that the reported results were limited to in vitro setups. Specifically, injecting the HAR products into ex vivo skin explants (e.g., abdominoplasty sections, porcine tissues) would further enable us to observe and compare their tissue integration properties. Additionally, semi-quantitative evaluation of collagen contents and quality via appropriate staining (e.g., Picrosirius Red, Masson’s Trichrome) would then be possible. A second technical limitation of the present study concerned the cell-based assays, which did not include orthogonal tests, notably for collagen quantification (e.g., by proteomics, ELISA). A third technical limitation concerned the low number of commercial comparators to the HAR gels, particularly cross-linked HA gels. Therein, alternative products from other well-established brands (e.g., Teoxane, Merz Aesthetics, or Vivacy) could have been used as comparators. Specifically, as the cross-linking techniques with BDDE are relatively similar, it would have been interesting to compare a hydrogel with a degree of cross-linking that is as close as possible to that of the HAR products.

Regarding future perspectives on this research, the HAR hydrogels could be injected subcutaneously in rats or intradermally in rabbits in order to monitor the in vivo mid-to-long-term performance of the products. Additionally, the HAR hydrogels could then be the subjects of a prospective clinical study, wherein cutaneous biopsies at various time-points would be performed on patients in order to quantify the collagen produced over time under real-world conditions. Finally, vitamin B3 could be formulated in alternative hydrogels in order to further study its intrinsic properties and effects from multi-parametric functional standpoints.

## 3. Conclusions

The results reported herein for the HAR-1 “Instant Refine” and HAR-3 “Maxi Lift” dermal fillers experimentally confirmed several functional impacts of vitamin B3 incorporation. The observed resilience of the HAR hydrogels (H_2_O_2_, AAPH, and xanthine oxidase assays) was interpreted as being highly beneficial from a post-administration system stability standpoint. Such attributes were specifically linked to the incorporation of vitamin B3 in the HA polymeric network, yielding a significant protective effect. Furthermore, the significant promotion of collagen production by dermal fibroblasts treated with HAR-1 and HAR-3 confirmed the intrinsic bio-stimulatory effects of both formulas, which differed from those of Radiesse^®^ and Sculptra™ comparators. The retained experimental methodologies of the study enabled to obtain characterization datasets of high translational relevance. Overall, the dual functionalization role of vitamin B3 in cross-linked HA-based dermal fillers was confirmed herein, providing a polyvalent treatment option (i.e., combined volumizer and bio-stimulant) for medical aesthetic professionals.

## 4. Materials and Methods

### 4.1. Reagents and Consumables Used for the Study

The reagents and consumables used in the present study are listed hereafter along with the corresponding manufacturers. The HAR-1 “Instant Refine” and HAR-3 “Maxi Lift” commercial dermal filler products were obtained from LOUNA AESTHETICS (Poisy, France). A 1 mL syringe of HAR-1 contained 15 mg of HA, 7.5 mg of vitamin B3, and 3 mg of lidocaine. A 1 mL syringe of HAR-3 contained 20 mg of HA, 7.5 mg of vitamin B3, and 3 mg of lidocaine. The initial HA in the HAR-1 and HAR-3 formulations had an intrinsic viscosity of 2.5 m^3^/kg. The JUVÉDERM^®^ VOLBELLA^®^ and VOLUMA^®^ products were purchased from Allergan Aesthetics (Annecy, France). The Radiesse^®^ product was purchased from Merz Aesthetics (Plan-les-Ouates, Switzerland). The Sculptra^™^ product was purchased from Galderma (Zug, Switzerland). Hyaluronidase from bovine testes (i.e., type VI-S), Collagen Assay Kits (i.e., reference MAK332), and BCA Protein Assay Kits were purchased from Sigma-Aldrich (St. Louis, MO, USA). Pharmaceutical-grade purified water and sterile phosphate-buffered saline (PBS) solutions were purchased from Laboratorium Dr. G. Bichsel (Unterseen, Switzerland). TrypLE™, DMEM, and FBS cell culture reagents were purchased from Life Technologies (Thermo Fisher Scientific, Waltham, MA, USA). Cell culture surfaces and disposable plastics were purchased from Greiner (Frickenhausen, Germany). Penicillin-streptomycin was obtained from the CHUV pharmacy (Lausanne, Switzerland). Except for the commercial analytical kits, all the reagents and consumables that were used in the study were procured in sterile form.

### 4.2. Hydrogel Rheological Characterization Method

In order to obtain quantitative and comparative information about important bio-physical attributes of the investigated hydrogels, a standard rheological setup was used. The basic rheological profiles of the selected commercial products were experimentally determined in oscillatory rheology using an HR 10 rheometer (TA Instruments, Guyancourt, France) equipped with a Peltier plate-plate. The storage modulus (G′), loss modulus (G″), and complex viscosity (η*) values of the samples were determined as a function of the applied oscillatory frequency. To this end, a frequency sweep was performed from 0.1 Hz to 10 Hz at a constant temperature of 25 °C. All measurements were then performed at 1 Hz and at 25 °C on volumes of 600 μL for the hydrogel samples and for the control groups. For the analyses, undiluted samples were dispensed on the baseplate of the rheometer and were submitted to controlled compression and shear stress by the instrument sensor plate. The shear stress was set at 3 N/m^2^ in all experiments to respect the linear viscoelastic region (LVE). Tan delta (tan δ) values were calculated from experimental G′ and G″ results (i.e., G″/G′ ratio). A sample hood was used during the rheological measurements to minimize sample water evaporation. The experimental storage moduli (G′) and loss moduli (G″) values of the samples were determined by the instrument using three experimental replicates for all of the assays. In detail, each replicate was obtained from a new hydrogel product syringe.

### 4.3. Hydrogel Accelerated Degradation Assays with Rheological Readouts

In order to study the breakdown of the hydrogel systems of interest (i.e., as a proxy for product resilience), various in vitro accelerated degradation assays were performed. The assays were specifically designed to compare the breakdown behaviors of the products in various setups. Firstly, the accelerated oxidative degradation of the included dermal fillers was assessed by means of rheology. Therefore, a volume of 300 µL of 30% H_2_O_2_, a volume of 300 µL of 2% 2,2’-azobis(2-amidinopropane) dihydrochloride (AAPH), or a volume of 300 µL of xanthine/xanthine oxidase mixture was added to 300 µL of the hydrogel sample. By direct contact, the various oxidant sources reacted with the samples and started to break down the polymer networks. The reaction tubes were then incubated at 37 °C under gentle shaking for 10 min before rheological readout. Following this standardized challenge period, the storage (G′) and loss (G″) moduli of the hydrogels were measured on an HR 10 rheometer (TA Instruments, Guyancourt, France) equipped with a Peltier plate-plate on 600 µL of sample. The constant oscillatory frequency was set at 1 Hz, and the assay temperature was set at 22 °C. The shear stress was set at 3 N/m^2^ in all experiments to respect the linear viscoelastic region (LVE). Tan delta (tan δ) values were calculated from experimental G′ and G″ results (i.e., G″/G′ ratio). A sample hood was used during the rheological measurements to minimize sample water evaporation. Three experimental replicates were used for all of the assays.

Secondly, the accelerated enzymatic degradation of the included dermal fillers was assessed by means of rheology. Therefore, a volume of 300 µL of 100 U/mL hyaluronidase was added to 300 µL of the hydrogel sample. By direct contact, the enzyme reacted with the samples and started to break down the polymer networks. The reaction tubes were then incubated at 37 °C under gentle shaking for 10 min before rheological readout. Following this standardized challenge period, the storage (G′) and loss (G″) moduli were measured on an HR 10 rheometer (TA Instruments, Guyancourt, France) equipped with a Peltier plate-plate on 600 µL of sample. The constant oscillatory frequency was set at 1 Hz, and the assay temperature was set at 22 °C. The shear stress was set at 3 N/m^2^ in all experiments to respect the linear viscoelastic region (LVE). A sample hood was used during the rheological measurements to minimize sample water evaporation. Three experimental replicates were used for all of the assays.

### 4.4. Hydrogel Cohesivity Analysis Method

In order to comparatively study the in situ behavior of the various hydrogels from another bio-physical attribute standpoint, the cohesiveness or cohesivity of the products was quantitatively assessed. Sample cohesivity attributes were determined using a standard drop-weight method. Therefore, the weight of an average fragment/drop of undiluted HA hydrogel was experimentally determined for each of the included dermal fillers using an 18 G or a 30 G needle. In detail, an automated setup was used to extrude the hydrogels drop-by-drop, in view of weighing each drop (i.e., ponderal proxy of system cohesivity). Therefore, the product syringe was mounted on the baseplate of a Texture Analyzer TA.XT. Plus instrument (Tracomme, Schlieren, Switzerland). The syringe plunger rod was automatically actuated by the instrument at a constant downward speed of 7.5 mm·min^−1^ or 12 mm·min^−1^. During this process, the hydrogels were automatically extruded into ambient air. When a constant extrusion force was attained (i.e., plateau extrusion force), at least 6 fragments/drops were collected and weighed to determine the average weight per drop. Three experimental replicates and three repetitions were used for all of the assays.

### 4.5. Hydrogel Injectability Analysis Method

In order to confirm that the considered hydrogels can be administered using standard medical equipment and normal operator strength, product injectability assays were performed. Standardized dermal filler injectability measurements were performed at a constant speed on a Texture Analyzer TA.XT. Plus instrument (Tracomme, Schlieren, Switzerland). An automated setup was used to continuously measure the force required for hydrogel product injection (i.e., the force that is applied on the plunger rod hilt). Therefore, the product syringe was mounted on the baseplate of the Texture Analyzer instrument. The injection force profiles of both HAR products were determined using the original syringes, with 30 G needles for HAR-1 and 27 G needles for HAR-3. The products were automatically injected by the instrument into a synthetic skin substitute (i.e., SimSkin^®^ from Wallcur™, San Diego, CA, USA) at a relatively fast and constant piston speed of 60 mm·min^−1^ at ambient temperature. Plateau injection forces were determined using force values acquired between 50% and 85% of the piston travel distance. Three experimental replicates were used for all of the assays. In detail, each replicate was obtained from a new hydrogel product syringe.

### 4.6. Product Biological Effect Evaluations in an In Vitro Dermal Fibroblast Model

#### 4.6.1. In Vitro Assessment of Cellular Viability/Metabolic Activity

In order to first assess the cytocompatibility/toxicity of the products of interest, these were incubated in direct contact with primary cells. In detail, primary dermal fibroblasts were retained for the study, as they are the most relevant target cellular population for dermal fillers (i.e., cells present in dermal tissues). Human primary dermal fibroblasts were grown in vitro in culture flasks containing Dulbecco’s modified Eagle medium (DMEM), supplemented with 10% fetal bovine serum (FBS) and 1% antibiotic–antimycotic solution. The cell cultures were incubated at 37 °C in a humidified atmosphere under 5% CO_2_ and were monitored daily using an inverted microscope. Subcultures were performed when a confluency level of 80% was observed. Therefore, the confluent fibroblasts were transferred to a 24-well plate (i.e., 10^4^ cells/well). After 24 h, the culture medium was replaced with a mix of hydrogel sample:medium at a 1:1 ratio. The cells were treated with the test-items for up to 5 days of incubation. Then, the adherent cells were rinsed and tested for viability/metabolic activity using the cell proliferation reagent WST-1 according to the specifications of the manufacturer. The assay was carried out four times, using four experimental replicates.

#### 4.6.2. In Vitro Assessment of Collagen Synthesis Stimulation Activity

In order to secondly assess the potential of the investigated products to stimulate collagen synthesis in the target cell population, endpoint quantifications of total collagen levels in the co-cultures (i.e., cell monolayers in contact with the test-items) were performed. For 96 h collagen quantification, the harvested cells were seeded into 24-well culture plates at a density of 10^5^ cells per well. After 24 h, the culture medium was replaced with 1000 µL of a mix of hydrogel sample:medium at a 1:1 ratio. The assay was carried out four times, using four experimental replicates. After 96 h of incubation, the supernatant was removed, and the wells were washed multiple times with PBS. The cells were then detached with TrypLE™ and hydrolyzed through cycles of freezing and thawing in order to measure the total collagen contents by fluorescence with the Collagen Assay Kit. Firstly, the collagen in the samples was enzymatically digested into peptides. Subsequently, the N-terminal glycine-containing peptides reacted with the dye reagent to form a fluorescent complex. The fluorescence intensity of this product, which was measured at λ_ex_ = 375/λ_em_ = 465 nm on a Varioskan™ LUX (Thermo Fisher Scientific, Waltham, MA, USA), was directly proportional to the total collagen concentration in the samples.

#### 4.6.3. In Vitro Assessment of Total Protein Synthesis Stimulation Activity

In order to thirdly assess the potential of the investigated products to stimulate protein synthesis in the target cell population, endpoint quantifications of total protein levels in the co-cultures (i.e., cell monolayers in contact with the test-items) were performed. Following the same procedure as for total collagen quantification, total protein concentrations after 96 h of incubation of the fibroblasts with the test-items were determined using a BCA assay kit. Bovine serum albumin (BSA) protein standards consisted of known concentrations of BSA and were prepared in a range of 200–1000 μg/mL. Following sample treatment with the assay reagent, the optical density at 562 nm was determined spectrophotometrically on a Varioskan™ LUX (Thermo Fisher Scientific, Waltham, MA, USA). The assay was carried out four times, using four experimental replicates.

### 4.7. Statistical Analyses and Data Presentation

The experimental data were reported herein as mean values and standard deviations, which were plotted as error bars in the graphs. For the statistical comparison of values from multi-group quantitative datasets, a one-way ANOVA test or a two-way ANOVA test was performed and was followed by a post hoc Tukey’s multiple comparison test. A *p*-value < 0.05 was retained as a general base for statistical significance determination. Detailed levels of statistical significance may be found in the Results section and in the Supplementary Tables. The statistical calculations and/or data presentation were performed using Microsoft Excel 365 (Microsoft Corporation, Redmond, WA, USA), Microsoft PowerPoint 365, and GraphPad Prism v.8.0.2 (GraphPad Software, San Diego, CA, USA).

## Figures and Tables

**Table 1:** Technical overview of the HA-based dermal filler products which were retained for experimental physico-mechanical characterization. Two JUVÉDERM^®^ reference products were included in the study as commercial comparators, based on finished product technical specification benchmarking [6]. G, gauge; HA, hyaluronic acid.

Product Commercial Name	Intended Product Uses ^1^	Needle Gauge ^2^ (G)	HA Concentration ^3^	Cross-Linker	Manufacturing Technology
HAR-1 “Instant Refine”	Fine lines; Peri-oral lines; Tear through	30 G	15 mg/mL	BDDE	Boost and Fusion
HAR-3 “Maxi Lift”	Volumizer	27 G	20 mg/mL	BDDE	Boost and Fusion
JUVÉDERM^®^ VOLBELLA^®^	Fine lines; Tear through	30 G	15 mg/mL	BDDE	VYCROSS^®^
JUVÉDERM^®^ VOLUMA^®^	Volumizer	27 G	20 mg/mL	BDDE	VYCROSS^®^

^1^ Product intended uses as specified by the manufacturers. ^2^ Needles as supplied with the commercial products. ^3^ HA contents as labeled on the products.

**Table 2:** Formulation details and selected hydrogel manufacturing process parameters for the HAR-1 and HAR-3 dermal fillers as reported by the manufacturer. HA, hyaluronic acid.

Product Parameters	Hydrogel Product	
	HAR-1	HAR-3
HA Concentration (mg/mL)	15.0	20.0
Vitamin B3 Concentration ^1^ (mg/mL)	7.50	7.50
Lidocaine Concentration (mg/mL)	3.00	3.00
Degree of HA Modification (%)	~3.8	~4.2

^1^ For both HAR products, the incorporation of vitamin B3 was performed in two steps.

**Table 3:** Injection force experimental values for the investigated HAR products at high injection speed (i.e., 1 mm·s^−1^). G, gauge; N, Newtons.

Product Name	Plateau Injection Force (N)	Maximum Injection Force (N)
HAR-1 “Instant Refine”	43.78 ± 0.51	44.62 ± 0.58
HAR-3 “Maxi Lift”	23.33 ± 0.65	23.49 ± 0.65
Empty Syringe Control ^1^	2.39 ± 0.13	2.48 ± 0.16

^1^ Empty syringes identical to those used for primary conditioning of the HAR products were used as controls.

**Table 4:** Technical description of the bio-stimulatory products included for biological effect characterization. CaHA, calcium hydroxylapatite; CMC, carboxymethylcellulose; HA, hyaluronic acid; PBS, phosphate-buffered saline; PLLA, poly-L-lactic acid.

Product Name	Intended Clinical Uses (Experimental Uses)	Product Conditioning ^1^ (Sample Preparation Method)	Main Product Composition	Ratio Prepared Product: Culture Medium ^2^
HAR-1 “Instant Refine”	Fine lines; Peri-oral lines; Tear through	1 mL syringe	HA; Vitamin B3	1:1
HAR-3 “Maxi Lift”	Volumizer	1 mL syringe	HA; Vitamin B3	1:1
Radiesse^®^	Moderate to severe facial wrinkles and folds; Lipoatrophy (Bio-stimulant control)	1.5 mL syringe (mixed with 6 mL water)	CaHA; CMC; Glycerin	1:1
Sculptra™	Volumizer; Lipoatrophy (Bio-stimulant control)	1 vial (lyophilizate resuspended in 5 mL water)	PLLA; CMC; Mannitol	1:1
JUVÉDERM^®^ VOLUMA^®^	Volumizer (Volumizer control)	1 mL syringe	HA	1:1
PBS	(Sham control)	500 mL bottle	Monopotassium phosphate; Disodium phosphate; Sodium chloride	1:1

^1^ Product conditioning unit as supplied by the manufacturers. ^2^ Proportion of each product in the cell-based assay, following the sample preparation step and sample dilution with equal volumes of culture medium.

## Data Availability

The data presented in this study are openly available within the article files.

## Supplementary Materials

The following supporting information can be downloaded at: https://www.mdpi.com/article/10.3390/gels10060361/s1, Figure S1: Supporting data for the rheological analyses; Figure S2: Supporting data for chemical mechanism investigation; Figure S3: Supporting data for the rheological analyses; Figure S4: Illustration of the investigated hydrogels; Figure S5: Supporting data for the injectability analyses; Figure S6: Illustration of the in vitro cell-based functional assay; Figure S7: Mechanistic explanation of the in vitro cell-based functional assay; Figure S8: Data illustrations from the in vitro cell-based functional assay; Table S1: Statistical analysis details; Table S2: Statistical analysis details; Table S3: Statistical analysis details; Table S4: Statistical analysis details; Table S5: Statistical analysis details; Table S6: Statistical analysis details; Table S7: Statistical analysis details; Table S8: Statistical analysis details; Video S1: Illustration of the drop-weight cohesivity determination setup.

## List of Abbreviations

AAPH	2,2′-azobis(2-amidinopropane) dihydrochloride
BCA	Bicinchoninic acid
BDDE	1,4-butanediol diglycidylether
BSA	Bovine serum albumin
CaHA	Calcium hydroxylapatite
CHUV	Lausanne University Hospital
CMC	Carboxymethylcellulose
DMEM	Dulbecco’s modified Eagle medium
ECM	Extracellular matrix
FBS	Fetal bovine serum
FDA	US Food and Drug Administration
G′	Storage modulus
G″	Loss modulus
HA	Hyaluronic acid
HAR	Boosting dermal fillers
HYAL	Hyaluronidase
H_2_O_2_	Hydrogen peroxide
LVE	Linear viscoelastic region
MD	Medical device
min	Minute
NA	Non-applicable
NMR	Nuclear magnetic resonance
NO	Nitric oxide
ns	Non-significant
Pa	Pascals
Pa·s	Pascal seconds
PBS	Phosphate-buffered saline
PLLA	Poly-l-lactic acid
ROS	Reactive oxygen species
s	Second
SEC-MS	Size-exclusion chromatography with mass spectrometry
USA	United States of America
UV	Ultraviolet
