# Supplementary material for: Dual Functionalization of Hyaluronan Dermal Fillers with Vitamin B3: Efficient Combination of Bio-Stimulation Properties with Hydrogel System Resilience Enhancement
Source: Gels. 2024 May 24;10(6):361. doi: 10.3390/gels10060361 (PMC11203111; doi:10.3390/gels10060361)
Supplement: Supplementary file 1 [file gels-10-00361-s001.zip › Gels-3013883 Supplementary Materials.pdf]

gels

MDPI

Supplementary Materials:

Dual Functionalization of Hyaluronan Dermal Fillers with Vitamin B3: Efficient Combination of Bio-Stimulation Properties with Hydrogel System Resilience Enhancement

Alexandre Porcello *, Michèle Chemali, Cíntia Marques, Corinne Scaletta, Kelly Lourenço, Philippe Abdel-Sayed, Wassim Raffoul, Nathalie Hirt-Burri, Lee Ann Applegate and Alexis Laurent *

1. Supplementary Figures

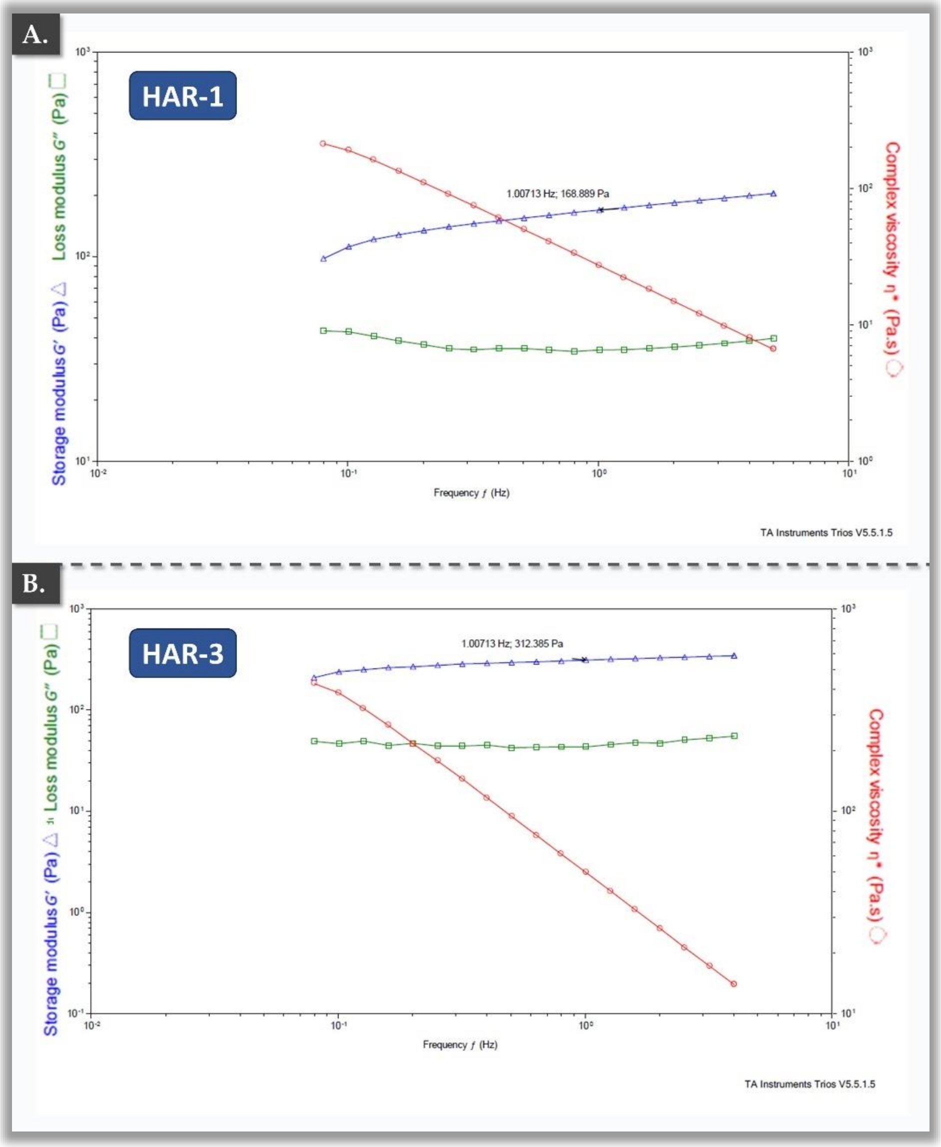

Figure S1. Rheological data complementing the results presented in Figure 1. (**A**) Storage modulus (G′), loss modulus (G′′), and complex viscosity (η*) curves of HAR-1 as a function of the applied oscillatory frequency. (**B**) Storage modulus (G′), loss modulus (G′′), and complex viscosity (η*) curves of HAR-3 as a function of the applied oscillatory frequency. Pa, Pascals; Pa·s, Pascal seconds.

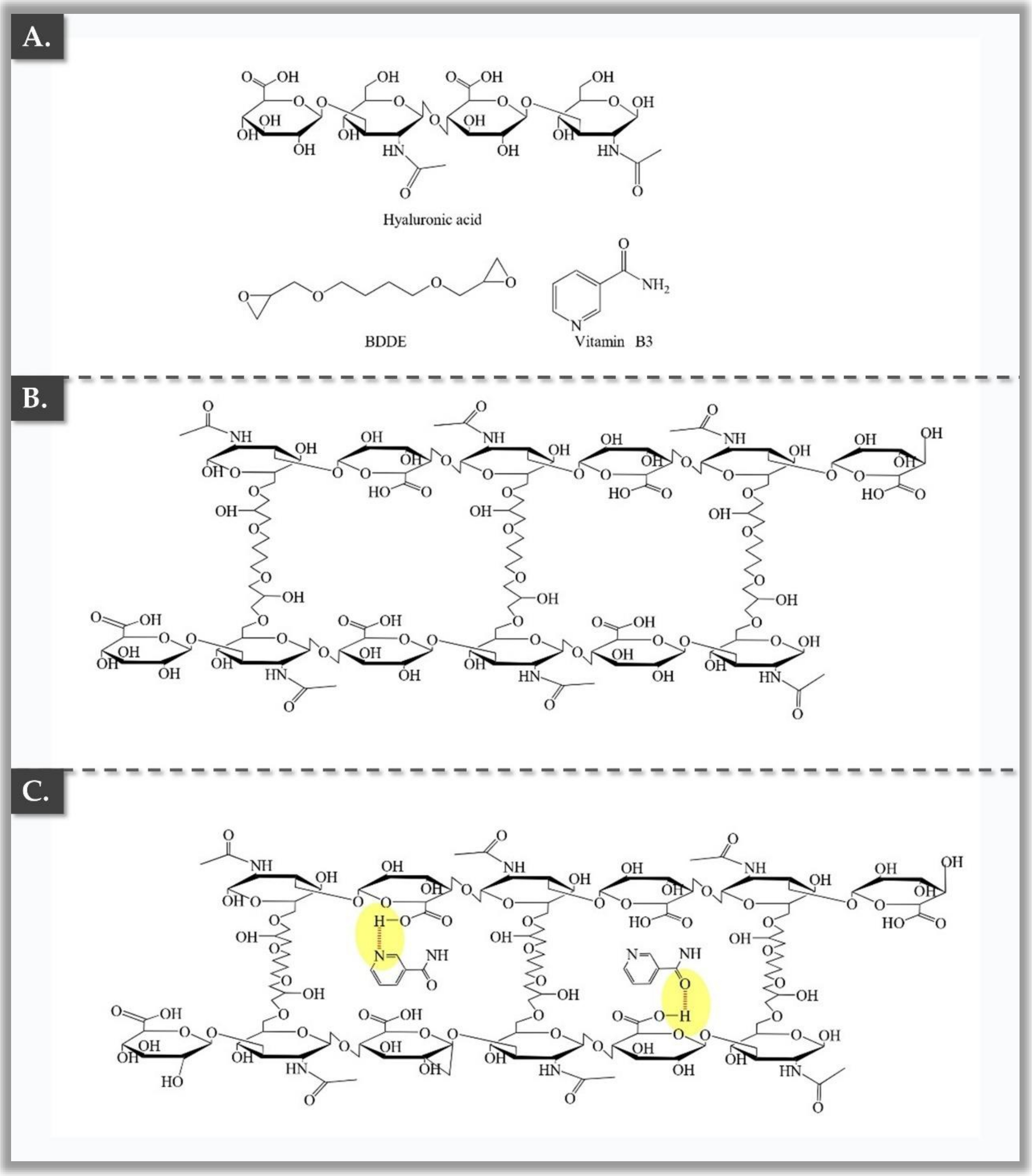

Figure S2. Illustration of the possible chemical interactions between vitamin B3 and BDDE-cross-linked HA. (**A**) Chemical structures of HA, BDDE, and vitamin B3. (**B**) Chemical structure of covalently BDDE-cross-linked HA. (**C**) Hypothesis of integration and interactions of vitamin B3 in the cross-linked HA polymeric network through hydrogen bonding. The N23 and O32 atoms of vitamin B3 (i.e., the nitrogen atom on the pyridine ring and the carbonyl oxygen atom on the amide group) are postulated as the main interaction sites with the hydrophilic groups of HA (i.e., including carboxylic acid groups). Potential interaction sites are evidenced in yellow. BDDE, 1,4-butanediol diglycidyl ether; HA, hyaluronic acid.

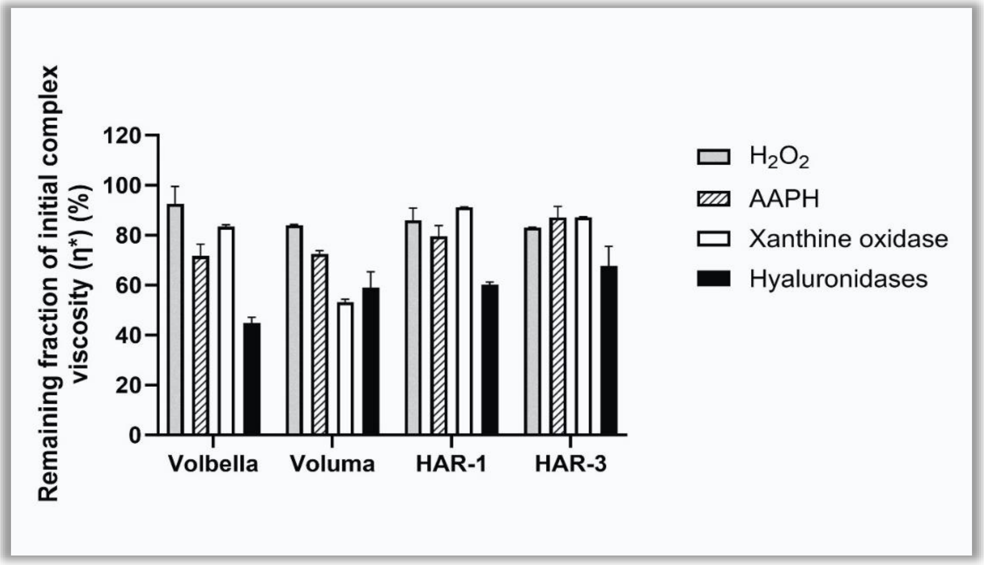

Figure S3. Rheological data complementing the results presented in Figure 2.

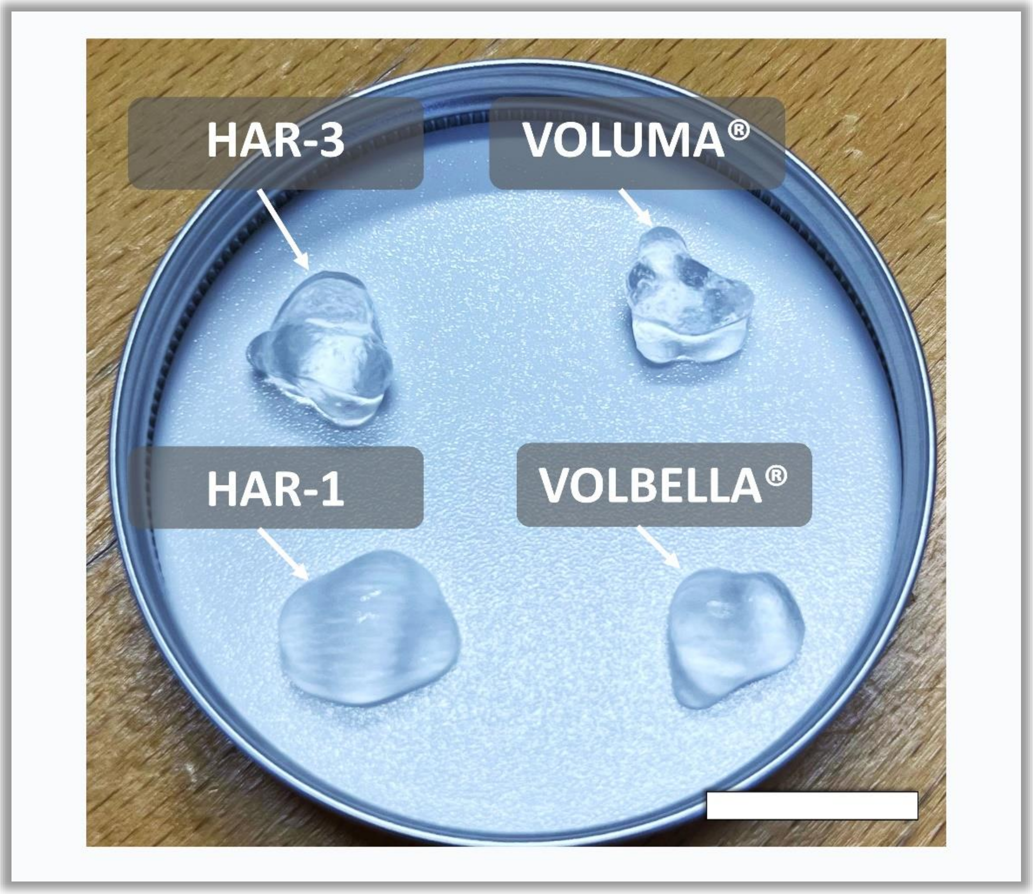

Figure S4. Photographic illustration of the various hydrogel products investigated in the study. Notable organoleptic similarities were respectively underscored between HAR-3 and VOLUMA® or between HAR-1 and VOLBELLA®, as expected. Scale bar = 10 mm.

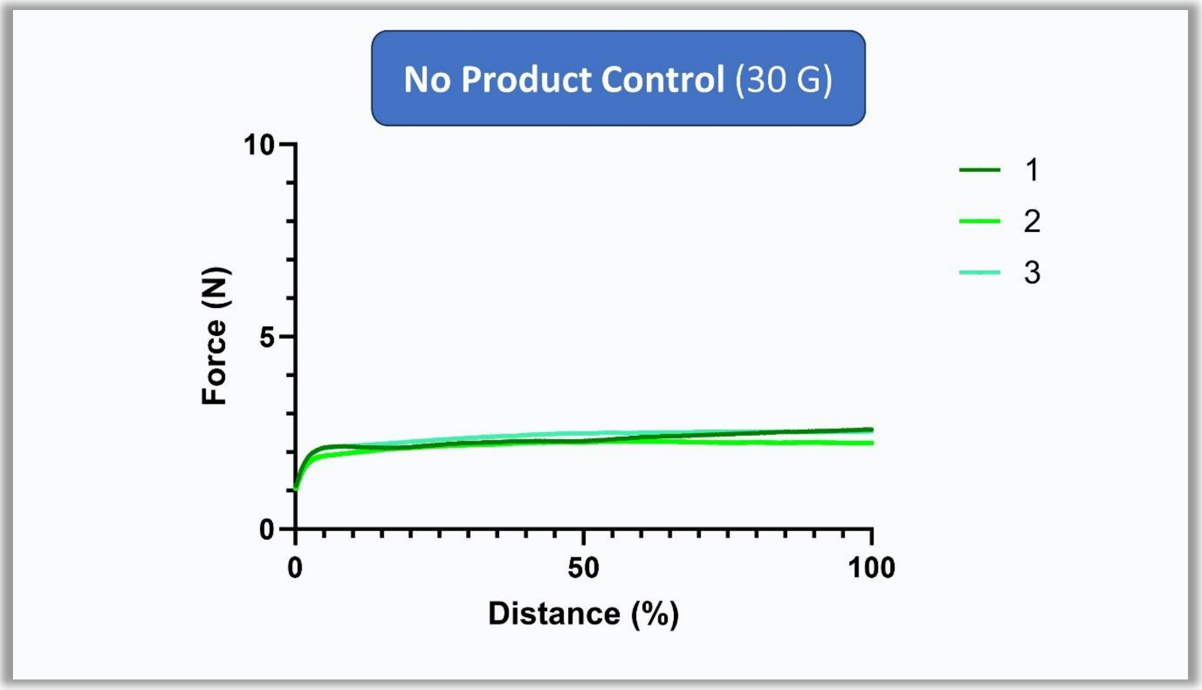

Figure S5. Injection force profiles of empty syringes (i.e., control group) in an automated measurement setup. Injectability results were presented for three distinct syringes, corresponding to profiles N°1, N°2, and N°3. Injectability assays were performed at ambient temperature using a constant plunger rod actuation speed of 1 mm·s^{-1}. N, Newtons.

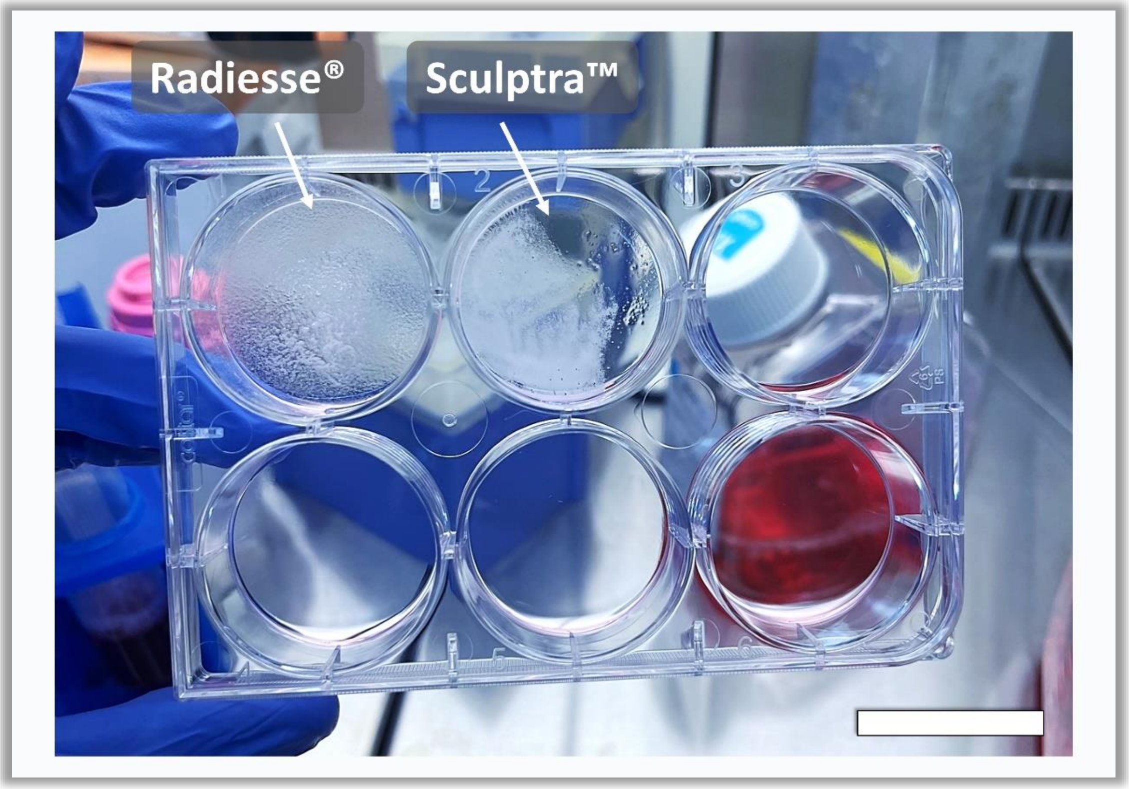

Figure S6. Photographic illustration of the cell-based assays presented in Figure 4 (i.e., experimental optimization phase in 6-well culture plates). The endpoint aspect of the reference bio-stimulators prior to cell monolayer rinsing is presented in wells A1 and A2. Scale bar = 40 mm.

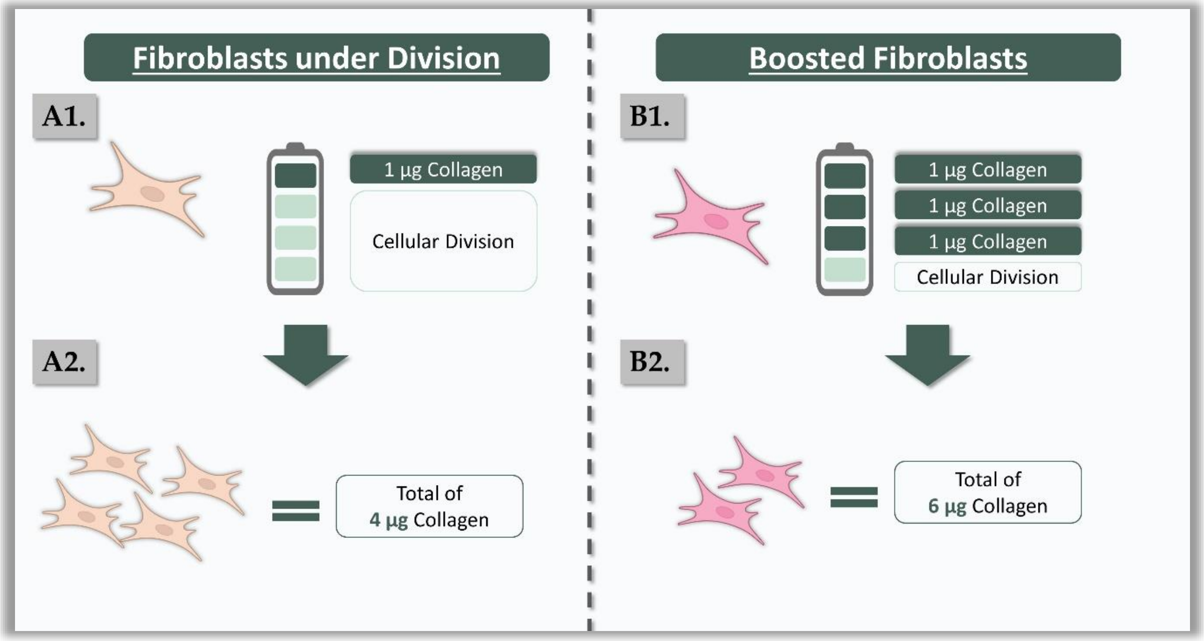

Figure S7. Schematic illustration of the effects of bio-stimulant treatment. (**A**) Cells treated with classical bio-stimulants (e.g., Radiesse®) multiply and use their energy to proliferate. One cell produces 1 µg of collagen (**A1**), thus four cells produce 4 µg of collagen (**A2**). (**B**) Cells treated with HAR products, using the Boost and Fusion technology, utilize vitamin B3 to produce more collagen. One cell produces 3 µg of collagen (**B1**), thus two cells produce 6 µg collagen (**B2**). HAR, Hyaluronic Acid-Reticulated.

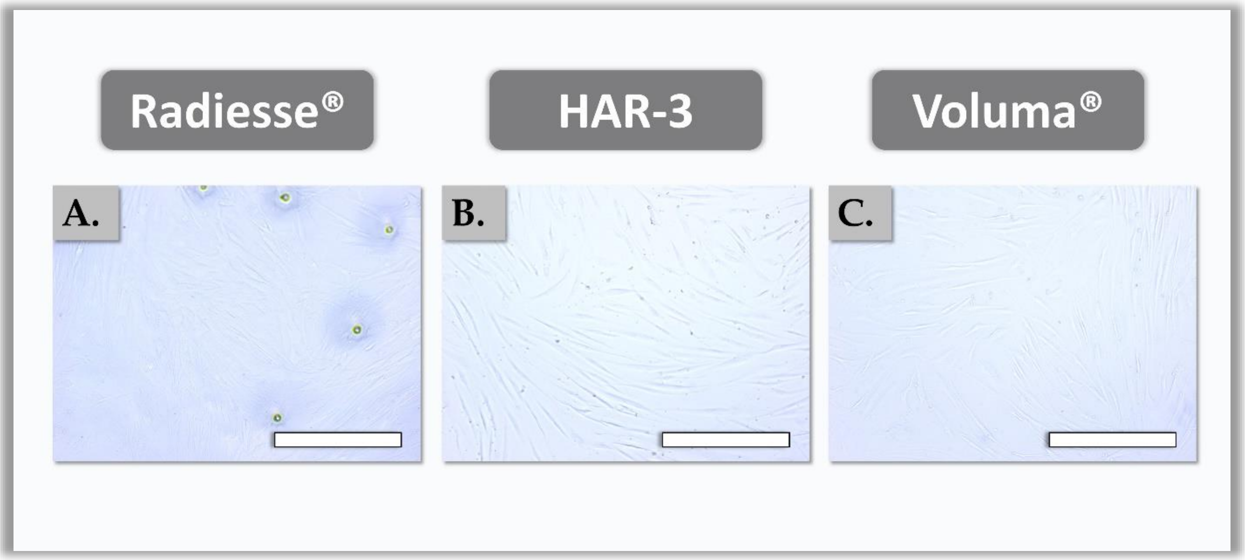

Figure S8. Photographic records (i.e., endpoint imaging) of the cell-based assays presented in Figure 4 (i.e., WST-1 cellular viability assessments, following in vitro direct contact with the samples). Scale bars = 75 μm.

2. Supplementary Tables

Table S1. Quantitative results of the post hoc Tukey's multiple comparison test, in relation with the comparative rheological data presented in Figure 1. Non-significant differences corresponded to a *p*-value > 0.05. ns, non-significant; Pa, Pascals.

Parameter	Compared Groups	Mean Absolute Difference	Adjusted *p*-Value	Significance Level [1]
Storage Modulus G′ (Pa)	VOLBELLA® vs. VOLUMA®	–97.92	< 0.0001	****
	VOLBELLA® vs. HAR-1	48.30	0.0006	***
	VOLBELLA® vs. HAR-3	–117.30	< 0.0001	****
	VOLUMA® vs. HAR-1	146.20	< 0.0001	****
	VOLUMA® vs. HAR-3	–19.38	0.0941	ns
	HAR-1 vs. HAR-3	–165.60	< 0.0001	****
Loss Modulus G″ (Pa)	VOLBELLA® vs. VOLUMA®	2.76	0.2085	ns
	VOLBELLA® vs. HAR-1	–2.49	0.2778	ns
	VOLBELLA® vs. HAR-3	–14.99	< 0.0001	****
	VOLUMA® vs. HAR-1	–5.25	0.0138	*
	VOLUMA® vs. HAR-3	–17.75	< 0.0001	****
	HAR-1 vs. HAR-3	–12.50	< 0.0001	****
Tan δ	VOLBELLA® vs. VOLUMA®	0.0549	< 0.0001	****
	VOLBELLA® vs. HAR-1	–0.0563	< 0.0001	****
	VOLBELLA® vs. HAR-3	0.0082	0.0972	ns
	VOLUMA® vs. HAR-1	–0.1112	< 0.0001	****
	VOLUMA® vs. HAR-3	–0.0467	< 0.0001	****
	HAR-1 vs. HAR-3	0.0645	< 0.0001	****

[1] A significance level described by one asterisk "*" corresponded to a *p*-value between 0.01 and 0.05. A significance level described by three asterisks "***" corresponded to a *p*-value between 0.0001 and 0.001. A significance level described by four asterisks "****" corresponded to a *p*-value inferior to 0.0001.

Table S2. Quantitative results of the post hoc Tukey's multiple comparison test, in relation with the comparative rheological data presented in Figure 2A (i.e., residual storage modulus G′ values in degradation assays). Non-significant differences corresponded to a *p*-value > 0.05. AAPH, 2,2'-azobis(2-amidinopropane) dihydrochloride; H_2O_2, hydrogen peroxide; ns, non-significant; Pa, Pascals.

Compared Groups	Mean Absolute Difference (%)	Adjusted *p*-Value	Significance Level [1]
VOLBELLA®:H_2O_2 vs. VOLBELLA®:AAPH	9.41	0.1940	ns
VOLBELLA®:H_2O_2 vs. VOLBELLA®:Xanthine oxidase	8.17	0.4048	ns
VOLBELLA®:H_2O_2 vs. VOLBELLA®:Hyaluronidases	27.92	< 0.0001	****
VOLBELLA®:H_2O_2 vs. VOLUMA®:H_2O_2	7.93	0.4571	ns
VOLBELLA®:H_2O_2 vs. VOLUMA®:AAPH	17.33	0.0187	*
VOLBELLA®:H_2O_2 vs. VOLUMA®:Xanthine oxidase	16.10	0.0415	*
VOLBELLA®:H_2O_2 vs. VOLUMA®:Hyaluronidases	35.84	< 0.0001	****
VOLBELLA®:H_2O_2 vs. HAR-1:H_2O_2	−3.94	0.9957	ns
VOLBELLA®:H_2O_2 vs. HAR-1:AAPH	5.47	0.9965	ns
VOLBELLA®:H_2O_2 vs. HAR-1:Xanthine oxidase	4.23	0.9998	ns
VOLBELLA®:H_2O_2 vs. HAR-1:Hyaluronidases	23.97	0.0001	***
VOLBELLA®:H_2O_2 vs. HAR-3:H_2O_2	−6.36	0.7931	ns
VOLBELLA®:H_2O_2 vs. HAR-3:AAPH	3.05	> 0.9999	ns
VOLBELLA®:H_2O_2 vs. HAR-3:Xanthine oxidase	1.82	> 0.9999	ns
VOLBELLA®:H_2O_2 vs. HAR-3:Hyaluronidases	21.56	0.0009	***
VOLBELLA®:AAPH vs. VOLBELLA®:Xanthine oxidase	−1.24	> 0.9999	ns
VOLBELLA®:AAPH vs. VOLBELLA®:Hyaluronidases	18.51	< 0.0001	****
VOLBELLA®:AAPH vs. VOLUMA®:H_2O_2	−1.49	> 0.9999	ns
VOLBELLA®:AAPH vs. VOLUMA®:AAPH	7.93	0.4571	ns
VOLBELLA®:AAPH vs. VOLUMA®:Xanthine oxidase	6.69	0.9758	ns
VOLBELLA®:AAPH vs. VOLUMA®:Hyaluronidases	26.43	< 0.0001	****
VOLBELLA®:AAPH vs. HAR-1:H_2O_2	−13.35	0.1899	ns
VOLBELLA®:AAPH vs. HAR-1:AAPH	−3.94	0.9957	ns
VOLBELLA®:AAPH vs. HAR-1:Xanthine oxidase	−5.18	0.9980	ns
VOLBELLA®:AAPH vs. HAR-1:Hyaluronidases	14.56	0.1018	ns
VOLBELLA®:AAPH vs. HAR-3:H_2O_2	−15.77	0.0508	ns
VOLBELLA®:AAPH vs. HAR-3:AAPH	−6.36	0.7931	ns
VOLBELLA®:AAPH vs. HAR-3:Xanthine oxidase	−7.60	0.9317	ns
VOLBELLA®:AAPH vs. HAR-3:Hyaluronidases	12.15	0.3230	ns
VOLBELLA®:Xanthine oxidase vs. VOLBELLA®:Hyaluronidases	19.74	< 0.0001	****
VOLBELLA®:Xanthine oxidase vs. VOLUMA®:H_2O_2	−0.25	> 0.9999	ns
VOLBELLA®:Xanthine oxidase vs. VOLUMA®:AAPH	9.16	0.7703	ns
VOLBELLA®:Xanthine oxidase vs. VOLUMA®:Xanthine oxidase	7.92	0.4571	ns
VOLBELLA®:Xanthine oxidase vs. VOLUMA®:Hyaluronidases	27.67	< 0.0001	****
VOLBELLA®:Xanthine oxidase vs. HAR-1:H_2O_2	−12.12	0.3275	ns
VOLBELLA®:Xanthine oxidase vs. HAR-1:AAPH	−2.71	> 0.9999	ns
VOLBELLA®:Xanthine oxidase vs. HAR-1:Xanthine oxidase	−3.94	0.9957	ns
VOLBELLA®:Xanthine oxidase vs. HAR-1:Hyaluronidases	15.80	0.0498	*
VOLBELLA®:Xanthine oxidase vs. HAR-3:H_2O_2	−14.53	0.1037	ns
VOLBELLA®:Xanthine oxidase vs. HAR-3:AAPH	−5.12	0.9983	ns

VOLBELLA®:Xanthine oxidase vs. HAR-3:Xanthine oxidase	−6.36	0.7931	ns
VOLBELLA®:Xanthine oxidase vs. HAR-3:Hyaluronidases	13.39	0.1868	ns
VOLBELLA®:Hyaluronidases vs. VOLUMA®:H_2O_2	−19.99	0.0028	**
VOLBELLA®:Hyaluronidases vs. VOLUMA®:AAPH	−10.58	0.5549	ns
VOLBELLA®:Hyaluronidases vs. VOLUMA®:Xanthine oxidase	−11.82	0.3674	ns
VOLBELLA®:Hyaluronidases vs. VOLUMA®:Hyaluronidases	7.93	0.4571	ns
VOLBELLA®:Hyaluronidases vs. HAR-1:H_2O_2	−31.86	< 0.0001	****
VOLBELLA®:Hyaluronidases vs. HAR-1:AAPH	−22.45	0.0004	***
VOLBELLA®:Hyaluronidases vs. HAR-1:Xanthine oxidase	−23.69	0.0002	***
VOLBELLA®:Hyaluronidases vs. HAR-1:Hyaluronidases	−3.94	0.9957	ns
VOLBELLA®:Hyaluronidases vs. HAR-3:H_2O_2	−34.27	< 0.0001	****
VOLBELLA®:Hyaluronidases vs. HAR-3:AAPH	−24.86	< 0.0001	****
VOLBELLA®:Hyaluronidases vs. HAR-3:Xanthine oxidase	−26.10	< 0.0001	****
VOLBELLA®:Hyaluronidases vs. HAR-3:Hyaluronidases	−6.36	0.7931	ns
VOLUMA®:H_2O_2 vs. VOLUMA®:AAPH	9.41	0.1940	ns
VOLUMA®:H_2O_2 vs. VOLUMA®:Xanthine oxidase	8.17	0.4048	ns
VOLUMA®:H_2O_2 vs. VOLUMA®:Hyaluronidases	27.92	< 0.0001	****
VOLUMA®:H_2O_2 vs. HAR-1:H_2O_2	−11.87	0.0269	*
VOLUMA®:H_2O_2 vs. HAR-1:AAPH	−2.46	> 0.9999	ns
VOLUMA®:H_2O_2 vs. HAR-1:Xanthine oxidase	−3.70	> 0.9999	ns
VOLUMA®:H_2O_2 vs. HAR-1:Hyaluronidases	16.05	0.0428	*
VOLUMA®:H_2O_2 vs. HAR-3:H_2O_2	−14.28	0.0024	**
VOLUMA®:H_2O_2 vs. HAR-3:AAPH	−4.87	0.9990	ns
VOLUMA®:H_2O_2 vs. HAR-3:Xanthine oxidase	−6.11	0.9894	ns
VOLUMA®:H_2O_2 vs. HAR-3:Hyaluronidases	13.64	0.1654	ns
VOLUMA®:AAPH vs. VOLUMA®:Xanthine oxidase	−1.24	> 0.9999	ns
VOLUMA®:AAPH vs. VOLUMA®:Hyaluronidases	18.51	< 0.0001	****
VOLUMA®:AAPH vs. HAR-1:H_2O_2	−21.28	0.0011	**
VOLUMA®:AAPH vs. HAR-1:AAPH	−11.87	0.0269	*
VOLUMA®:AAPH vs. HAR-1:Xanthine oxidase	−13.10	0.2134	ns
VOLUMA®:AAPH vs. HAR-1:Hyaluronidases	6.64	0.9772	ns
VOLUMA®:AAPH vs. HAR-3:H_2O_2	−23.69	0.0002	***
VOLUMA®:AAPH vs. HAR-3:AAPH	−14.28	0.0024	**
VOLUMA®:AAPH vs. HAR-3:Xanthine oxidase	−15.52	0.0590	ns
VOLUMA®:AAPH vs. HAR-3:Hyaluronidases	4.23	0.9998	ns
VOLUMA®:Xanthine oxidase vs. VOLUMA®:Hyaluronidases	19.74	< 0.0001	****
VOLUMA®:Xanthine oxidase vs. HAR-1:H_2O_2	−20.04	0.0027	**
VOLUMA®:Xanthine oxidase vs. HAR-1:AAPH	−10.63	0.5473	ns
VOLUMA®:Xanthine oxidase vs. HAR-1:Xanthine oxidase	−11.87	0.0269	*
VOLUMA®:Xanthine oxidase vs. HAR-1:Hyaluronidases	7.88	0.9109	ns
VOLUMA®:Xanthine oxidase vs. HAR-3:H_2O_2	−22.45	0.0004	***
VOLUMA®:Xanthine oxidase vs. HAR-3:AAPH	−13.04	0.2195	ns

VOLUMA®:Xanthine oxidase vs. HAR-3:Xanthine oxidase	–14.28	0.0024	**
VOLUMA®:Xanthine oxidase vs. HAR-3:Hyaluronidases	5.46	0.9966	ns
VOLUMA®:Hyaluronidases vs. HAR-1:H_2O_2	–39.78	< 0.0001	****
VOLUMA®:Hyaluronidases vs. HAR-1:AAPH	–30.37	< 0.0001	****
VOLUMA®:Hyaluronidases vs. HAR-1:Xanthine oxidase	–31.61	< 0.0001	****
VOLUMA®:Hyaluronidases vs. HAR-1:Hyaluronidases	–11.87	0.0269	*
VOLUMA®:Hyaluronidases vs. HAR-3:H_2O_2	–42.20	< 0.0001	****
VOLUMA®:Hyaluronidases vs. HAR-3:AAPH	–32.79	< 0.0001	****
VOLUMA®:Hyaluronidases vs. HAR-3:Xanthine oxidase	–34.03	< 0.0001	****
VOLUMA®:Hyaluronidases vs. HAR-3:Hyaluronidases	–14.28	0.0024	**
HAR-1:H_2O_2 vs. HAR-1:AAPH	9.41	0.1940	ns
HAR-1:H_2O_2 vs. HAR-1:Xanthine oxidase	8.17	0.4048	ns
HAR-1:H_2O_2 vs. HAR-1:Hyaluronidases	27.92	< 0.0001	****
HAR-1:H_2O_2 vs. HAR-3:H_2O_2	–2.41	> 0.9999	ns
HAR-1:H_2O_2 vs. HAR-3:AAPH	6.99	0.9644	ns
HAR-1:H_2O_2 vs. HAR-3:Xanthine oxidase	5.76	0.9941	ns
HAR-1:H_2O_2 vs. HAR-3:Hyaluronidases	25.50	< 0.0001	****
HAR-1:AAPH vs. HAR-1:Xanthine oxidase	–1.24	> 0.9999	ns
HAR-1:AAPH vs. HAR-1:Hyaluronidases	18.51	< 0.0001	****
HAR-1:AAPH vs. HAR-3:H_2O_2	–11.82	0.3668	ns
HAR-1:AAPH vs. HAR-3:AAPH	–2.41	> 0.9999	ns
HAR-1:AAPH vs. HAR-3:Xanthine oxidase	–3.65	> 0.9999	ns
HAR-1:AAPH vs. HAR-3:Hyaluronidases	16.09	0.0416	*
HAR-1:Xanthine oxidase vs. HAR-1:Hyaluronidases	19.74	< 0.0001	****
HAR-1:Xanthine oxidase vs. HAR-3:H_2O_2	–10.59	0.5542	ns
HAR-1:Xanthine oxidase vs. HAR-3:AAPH	–1.18	> 0.9999	ns
HAR-1:Xanthine oxidase vs. HAR-3:Xanthine oxidase	–2.41	> 0.9999	ns
HAR-1:Xanthine oxidase vs. HAR-3:Hyaluronidases	17.33	0.0188	*
HAR-1:Hyaluronidases vs. HAR-3:H_2O_2	–30.33	< 0.0001	****
HAR-1:Hyaluronidases vs. HAR-3:AAPH	–20.92	0.0014	**
HAR-1:Hyaluronidases vs. HAR-3:Xanthine oxidase	–22.16	0.0005	***
HAR-1:Hyaluronidases vs. HAR-3:Hyaluronidases	–2.41	> 0.9999	ns
HAR-3:H_2O_2 vs. HAR-3:AAPH	9.41	0.1940	ns
HAR-3:H_2O_2 vs. HAR-3:Xanthine oxidase	8.17	0.4048	ns
HAR-3:H_2O_2 vs. HAR-3:Hyaluronidases	27.92	< 0.0001	****
HAR-3:AAPH vs. HAR-3:Xanthine oxidase	–1.24	> 0.9999	ns
HAR-3:AAPH vs. HAR-3:Hyaluronidases	18.51	< 0.0001	****
HAR-3:Xanthine oxidase vs. HAR-3:Hyaluronidases	19.74	< 0.0001	****

[1] A significance level described by one asterisk "*" corresponded to a *p*-value between 0.01 and 0.05. A significance level described by two asterisks "**" corresponded to a *p*-value between 0.001 and 0.01. A significance level described by three asterisks "***" corresponded to a *p*-value between 0.0001 and 0.001. A significance level described by four asterisks "****" corresponded to a *p*-value inferior to 0.0001.

Table S3. Quantitative results of the post hoc Tukey's multiple comparison test, in relation with the comparative rheological data presented in Figure 2B (i.e., residual loss modulus G'' values in degradation assays). Non-significant differences corresponded to a *p*-value > 0.05. AAPH, 2,2'-azobis(2-amidinopropane) dihydrochloride; H_2O_2, hydrogen peroxide; ns, non-significant; Pa, Pascals.

**Compared Groups**	**Mean Absolute Difference** (%)	**Adjusted *p*-Value**	**Significance Level** [1]
VOLBELLA®:H_2O_2 vs. VOLBELLA®:AAPH	6.05	0.9021	ns
VOLBELLA®:H_2O_2 vs. VOLBELLA®:Xanthine oxidase	−11.81	0.0623	ns
VOLBELLA®:H_2O_2 vs. VOLBELLA®:Hyaluronidases	47.46	< 0.0001	****
VOLBELLA®:H_2O_2 vs. VOLUMA®:H_2O_2	−25.54	< 0.0001	****
VOLBELLA®:H_2O_2 vs. VOLUMA®:AAPH	−52.24	< 0.0001	****
VOLBELLA®:H_2O_2 vs. VOLUMA®:Xanthine oxidase	−6.99	0.7645	ns
VOLBELLA®:H_2O_2 vs. VOLUMA®:Hyaluronidases	41.38	< 0.0001	****
VOLBELLA®:H_2O_2 vs. HAR-1:H_2O_2	−33.18	< 0.0001	****
VOLBELLA®:H_2O_2 vs. HAR-1:AAPH	−42.35	< 0.0001	****
VOLBELLA®:H_2O_2 vs. HAR-1:Xanthine oxidase	−46.08	< 0.0001	****
VOLBELLA®:H_2O_2 vs. HAR-1:Hyaluronidases	30.50	< 0.0001	****
VOLBELLA®:H_2O_2 vs. HAR-3:H_2O_2	−31.32	< 0.0001	****
VOLBELLA®:H_2O_2 vs. HAR-3:AAPH	−36.70	< 0.0001	****
VOLBELLA®:H_2O_2 vs. HAR-3:Xanthine oxidase	−29.22	< 0.0001	****
VOLBELLA®:H_2O_2 vs. HAR-3:Hyaluronidases	13.87	0.0116	*
VOLBELLA®:AAPH vs. VOLBELLA®:Xanthine oxidase	−17.86	0.0003	***
VOLBELLA®:AAPH vs. VOLBELLA®:Hyaluronidases	41.41	< 0.0001	****
VOLBELLA®:AAPH vs. VOLUMA®:H_2O_2	−31.59	< 0.0001	****
VOLBELLA®:AAPH vs. VOLUMA®:AAPH	−58.29	< 0.0001	****
VOLBELLA®:AAPH vs. VOLUMA®:Xanthine oxidase	−13.04	0.0236	*
VOLBELLA®:AAPH vs. VOLUMA®:Hyaluronidases	35.33	< 0.0001	****
VOLBELLA®:AAPH vs. HAR-1:H_2O_2	−39.23	< 0.0001	****
VOLBELLA®:AAPH vs. HAR-1:AAPH	−48.39	< 0.0001	****
VOLBELLA®:AAPH vs. HAR-1:Xanthine oxidase	−52.13	< 0.0001	****
VOLBELLA®:AAPH vs. HAR-1:Hyaluronidases	24.45	< 0.0001	****
VOLBELLA®:AAPH vs. HAR-3:H_2O_2	−37.37	< 0.0001	****
VOLBELLA®:AAPH vs. HAR-3:AAPH	−42.75	< 0.0001	****
VOLBELLA®:AAPH vs. HAR-3:Xanthine oxidase	−35.27	< 0.0001	****
VOLBELLA®:AAPH vs. HAR-3:Hyaluronidases	7.82	0.6013	ns
VOLBELLA®:Xanthine oxidase vs. VOLBELLA®:Hyaluronidases	59.27	< 0.0001	****
VOLBELLA®:Xanthine oxidase vs. VOLUMA®:H_2O_2	−13.73	0.0131	*
VOLBELLA®:Xanthine oxidase vs. VOLUMA®:AAPH	−40.43	< 0.0001	****
VOLBELLA®:Xanthine oxidase vs. VOLUMA®:Xanthine oxidase	4.83	0.9841	ns
VOLBELLA®:Xanthine oxidase vs. VOLUMA®:Hyaluronidases	53.19	< 0.0001	****
VOLBELLA®:Xanthine oxidase vs. HAR-1:H_2O_2	−21.37	< 0.0001	****
VOLBELLA®:Xanthine oxidase vs. HAR-1:AAPH	−30.53	< 0.0001	****
VOLBELLA®:Xanthine oxidase vs. HAR-1:Xanthine oxidase	−34.26	< 0.0001	****
VOLBELLA®:Xanthine oxidase vs. HAR-1:Hyaluronidases	42.31	< 0.0001	****
VOLBELLA®:Xanthine oxidase vs. HAR-3:H_2O_2	−19.51	< 0.0001	****
VOLBELLA®:Xanthine oxidase vs. HAR-3:AAPH	−24.89	< 0.0001	****

VOLBELLA®:Xanthine oxidase vs. HAR-3:Xanthine oxidase	–17.41	0.0004	***
VOLBELLA®:Xanthine oxidase vs. HAR-3:Hyaluronidases	25.68	< 0.0001	****
VOLBELLA®:Hyaluronidases vs. VOLUMA®:H_2O_2	–73.00	< 0.0001	****
VOLBELLA®:Hyaluronidases vs. VOLUMA®:AAPH	–99.71	< 0.0001	****
VOLBELLA®:Hyaluronidases vs. VOLUMA®:Xanthine oxidase	–54.45	< 0.0001	****
VOLBELLA®:Hyaluronidases vs. VOLUMA®:Hyaluronidases	–6.08	0.8982	ns
VOLBELLA®:Hyaluronidases vs. HAR-1:H_2O_2	–80.64	< 0.0001	****
VOLBELLA®:Hyaluronidases vs. HAR-1:AAPH	–89.81	< 0.0001	****
VOLBELLA®:Hyaluronidases vs. HAR-1:Xanthine oxidase	–93.54	< 0.0001	****
VOLBELLA®:Hyaluronidases vs. HAR-1:Hyaluronidases	–16.96	0.0006	***
VOLBELLA®:Hyaluronidases vs. HAR-3:H_2O_2	–78.79	< 0.0001	****
VOLBELLA®:Hyaluronidases vs. HAR-3:AAPH	–84.17	< 0.0001	****
VOLBELLA®:Hyaluronidases vs. HAR-3:Xanthine oxidase	–76.68	< 0.0001	****
VOLBELLA®:Hyaluronidases vs. HAR-3:Hyaluronidases	–33.59	< 0.0001	****
VOLUMA®:H_2O_2 vs. VOLUMA®:AAPH	–26.71	< 0.0001	****
VOLUMA®:H_2O_2 vs. VOLUMA®:Xanthine oxidase	18.55	0.0001	***
VOLUMA®:H_2O_2 vs. VOLUMA®:Hyaluronidases	66.92	< 0.0001	****
VOLUMA®:H_2O_2 vs. HAR-1:H_2O_2	–7.64	0.6384	ns
VOLUMA®:H_2O_2 vs. HAR-1:AAPH	–16.81	0.0007	***
VOLUMA®:H_2O_2 vs. HAR-1:Xanthine oxidase	–20.54	< 0.0001	****
VOLUMA®:H_2O_2 vs. HAR-1:Hyaluronidases	56.04	< 0.0001	****
VOLUMA®:H_2O_2 vs. HAR-3:H_2O_2	–5.79	0.9286	ns
VOLUMA®:H_2O_2 vs. HAR-3:AAPH	–11.17	0.0998	ns
VOLUMA®:H_2O_2 vs. HAR-3:Xanthine oxidase	–3.68	0.9990	ns
VOLUMA®:H_2O_2 vs. HAR-3:Hyaluronidases	39.41	< 0.0001	****
VOLUMA®:AAPH vs. VOLUMA®:Xanthine oxidase	45.26	< 0.0001	****
VOLUMA®:AAPH vs. VOLUMA®:Hyaluronidases	93.62	< 0.0001	****
VOLUMA®:AAPH vs. HAR-1:H_2O_2	19.07	< 0.0001	****
VOLUMA®:AAPH vs. HAR-1:AAPH	9.90	0.2268	ns
VOLUMA®:AAPH vs. HAR-1:Xanthine oxidase	6.17	0.8883	ns
VOLUMA®:AAPH vs. HAR-1:Hyaluronidases	82.74	< 0.0001	****
VOLUMA®:AAPH vs. HAR-3:H_2O_2	20.92	< 0.0001	****
VOLUMA®:AAPH vs. HAR-3:AAPH	15.54	0.0025	**
VOLUMA®:AAPH vs. HAR-3:Xanthine oxidase	23.02	< 0.0001	****
VOLUMA®:AAPH vs. HAR-3:Hyaluronidases	66.11	< 0.0001	****
VOLUMA®:Xanthine oxidase vs. VOLUMA®:Hyaluronidases	48.37	< 0.0001	****
VOLUMA®:Xanthine oxidase vs. HAR-1:H_2O_2	–26.19	< 0.0001	****
VOLUMA®:Xanthine oxidase vs. HAR-1:AAPH	–35.36	< 0.0001	****
VOLUMA®:Xanthine oxidase vs. HAR-1:Xanthine oxidase	–39.09	< 0.0001	****
VOLUMA®:Xanthine oxidase vs. HAR-1:Hyaluronidases	37.49	< 0.0001	****
VOLUMA®:Xanthine oxidase vs. HAR-3:H_2O_2	–24.34	< 0.0001	****
VOLUMA®:Xanthine oxidase vs. HAR-3:AAPH	–29.72	< 0.0001	****

VOLUMA®:Xanthine oxidase vs. HAR-3:Xanthine oxidase	–22.23	< 0.0001	****
VOLUMA®:Xanthine oxidase vs. HAR-3:Hyaluronidases	20.86	< 0.0001	****
VOLUMA®:Hyaluronidases vs. HAR-1:H_2O_2	–74.56	< 0.0001	****
VOLUMA®:Hyaluronidases vs. HAR-1:AAPH	–83.73	< 0.0001	****
VOLUMA®:Hyaluronidases vs. HAR-1:Xanthine oxidase	–87.46	< 0.0001	****
VOLUMA®:Hyaluronidases vs. HAR-1:Hyaluronidases	–10.88	0.1216	ns
VOLUMA®:Hyaluronidases vs. HAR-3:H_2O_2	–72.70	< 0.0001	****
VOLUMA®:Hyaluronidases vs. HAR-3:AAPH	–78.08	< 0.0001	****
VOLUMA®:Hyaluronidases vs. HAR-3:Xanthine oxidase	–70.60	< 0.0001	****
VOLUMA®:Hyaluronidases vs. HAR-3:Hyaluronidases	–27.51	< 0.0001	****
HAR-1:H_2O_2 vs. HAR-1:AAPH	–9.17	0.3384	ns
HAR-1:H_2O_2 vs. HAR-1:Xanthine oxidase	–12.90	0.0264	*
HAR-1:H_2O_2 vs. HAR-1:Hyaluronidases	63.68	< 0.0001	****
HAR-1:H_2O_2 vs. HAR-3:H_2O_2	1.85	> 0.9999	ns
HAR-1:H_2O_2 vs. HAR-3:AAPH	–3.53	0.9994	ns
HAR-1:H_2O_2 vs. HAR-3:Xanthine oxidase	3.96	0.9978	ns
HAR-1:H_2O_2 vs. HAR-3:Hyaluronidases	47.05	< 0.0001	****
HAR-1:AAPH vs. HAR-1:Xanthine oxidase	–3.73	0.9988	ns
HAR-1:AAPH vs. HAR-1:Hyaluronidases	72.85	< 0.0001	****
HAR-1:AAPH vs. HAR-3:H_2O_2	11.02	0.1103	ns
HAR-1:AAPH vs. HAR-3:AAPH	5.64	0.9409	ns
HAR-1:AAPH vs. HAR-3:Xanthine oxidase	13.13	0.0219	*
HAR-1:AAPH vs. HAR-3:Hyaluronidases	56.22	< 0.0001	****
HAR-1:Xanthine oxidase vs. HAR-1:Hyaluronidases	76.58	< 0.0001	****
HAR-1:Xanthine oxidase vs. HAR-3:H_2O_2	14.75	0.0052	**
HAR-1:Xanthine oxidase vs. HAR-3:AAPH	9.37	0.3043	ns
HAR-1:Xanthine oxidase vs. HAR-3:Xanthine oxidase	16.86	0.0007	***
HAR-1:Xanthine oxidase vs. HAR-3:Hyaluronidases	59.95	< 0.0001	****
HAR-1:Hyaluronidases vs. HAR-3:H_2O_2	–61.82	< 0.0001	****
HAR-1:Hyaluronidases vs. HAR-3:AAPH	–67.20	< 0.0001	****
HAR-1:Hyaluronidases vs. HAR-3:Xanthine oxidase	–59.72	< 0.0001	****
HAR-1:Hyaluronidases vs. HAR-3:Hyaluronidases	–16.63	0.0009	***
HAR-3:H_2O_2 vs. HAR-3:AAPH	–5.38	0.9594	ns
HAR-3:H_2O_2 vs. HAR-3:Xanthine oxidase	2.10	> 0.9999	ns
HAR-3:H_2O_2 vs. HAR-3:Hyaluronidases	45.19	< 0.0001	****
HAR-3:AAPH vs. HAR-3:Xanthine oxidase	7.48	0.6700	ns
HAR-3:AAPH vs. HAR-3:Hyaluronidases	50.57	< 0.0001	****
HAR-3:Xanthine oxidase vs. HAR-3:Hyaluronidases	43.09	< 0.0001	****

[1] A significance level described by one asterisk "*" corresponded to a *p*-value between 0.01 and 0.05. A significance level described by two asterisks "**" corresponded to a *p*-value between 0.001 and 0.01. A significance level described by three asterisks "***" corresponded to a *p*-value between 0.0001 and 0.001. A significance level described by four asterisks "****" corresponded to a *p*-value inferior to 0.0001.

Table S4. Quantitative results of the post hoc Tukey's multiple comparison test, in relation with the comparative rheological data presented in Figure S3 (i.e., residual complex viscosity η* values in degradation assays). Non-significant differences corresponded to a *p*-value > 0.05. AAPH, 2,2'-azobis(2-amidinopropane) dihydrochloride; H_2O_2, hydrogen peroxide; ns, non-significant; Pa, Pascals.

**Compared Groups**	**Mean Absolute Difference** (%)	**Adjusted *p*-Value**	**Significance Level** [1]
VOLBELLA®:H_2O_2 vs. VOLBELLA®:AAPH	20.84	< 0.0001	****
VOLBELLA®:H_2O_2 vs. VOLBELLA®:Xanthine oxidase	9.09	0.1134	ns
VOLBELLA®:H_2O_2 vs. VOLBELLA®:Hyaluronidases	47.71	< 0.0001	****
VOLBELLA®:H_2O_2 vs. VOLUMA®:H_2O_2	8.52	0.1779	ns
VOLBELLA®:H_2O_2 vs. VOLUMA®:AAPH	19.94	< 0.0001	****
VOLBELLA®:H_2O_2 vs. VOLUMA®:Xanthine oxidase	39.39	< 0.0001	****
VOLBELLA®:H_2O_2 vs. VOLUMA®:Hyaluronidases	33.38	< 0.0001	****
VOLBELLA®:H_2O_2 vs. HAR-1:H_2O_2	6.68	0.5485	ns
VOLBELLA®:H_2O_2 vs. HAR-1:AAPH	13.02	0.0021	**
VOLBELLA®:H_2O_2 vs. HAR-1:Xanthine oxidase	1.39	> 0.9999	ns
VOLBELLA®:H_2O_2 vs. HAR-1:Hyaluronidases	32.42	< 0.0001	****
VOLBELLA®:H_2O_2 vs. HAR-3:H_2O_2	9.34	0.0914	ns
VOLBELLA®:H_2O_2 vs. HAR-3:AAPH	5.42	0.8371	ns
VOLBELLA®:H_2O_2 vs. HAR-3:Xanthine oxidase	5.32	0.8539	ns
VOLBELLA®:H_2O_2 vs. HAR-3:Hyaluronidases	24.73	< 0.0001	****
VOLBELLA®:AAPH vs. VOLBELLA®:Xanthine oxidase	−11.76	0.0086	**
VOLBELLA®:AAPH vs. VOLBELLA®:Hyaluronidases	26.87	< 0.0001	****
VOLBELLA®:AAPH vs. VOLUMA®:H_2O_2	−12.32	0.0046	**
VOLBELLA®:AAPH vs. VOLUMA®:AAPH	−0.90	> 0.9999	ns
VOLBELLA®:AAPH vs. VOLUMA®:Xanthine oxidase	18.55	< 0.0001	****
VOLBELLA®:AAPH vs. VOLUMA®:Hyaluronidases	12.54	0.0036	**
VOLBELLA®:AAPH vs. HAR-1:H_2O_2	−14.16	0.0006	***
VOLBELLA®:AAPH vs. HAR-1:AAPH	−7.83	0.2904	ns
VOLBELLA®:AAPH vs. HAR-1:Xanthine oxidase	−19.45	< 0.0001	****
VOLBELLA®:AAPH vs. HAR-1:Hyaluronidases	11.57	0.0104	*
VOLBELLA®:AAPH vs. HAR-3:H_2O_2	−11.50	0.0112	*
VOLBELLA®:AAPH vs. HAR-3:AAPH	−15.43	0.0001	***
VOLBELLA®:AAPH vs. HAR-3:Xanthine oxidase	−15.52	0.0001	***
VOLBELLA®:AAPH vs. HAR-3:Hyaluronidases	3.89	0.9876	ns
VOLBELLA®:Xanthine oxidase vs. VOLBELLA®:Hyaluronidases	38.63	< 0.0001	****
VOLBELLA®:Xanthine oxidase vs. VOLUMA®:H_2O_2	−0.57	> 0.9999	ns
VOLBELLA®:Xanthine oxidase vs. VOLUMA®:AAPH	10.86	0.0220	*
VOLBELLA®:Xanthine oxidase vs. VOLUMA®:Xanthine oxidase	30.31	< 0.0001	****
VOLBELLA®:Xanthine oxidase vs. VOLUMA®:Hyaluronidases	24.30	< 0.0001	****
VOLBELLA®:Xanthine oxidase vs. HAR-1:H_2O_2	−2.40	> 0.9999	ns
VOLBELLA®:Xanthine oxidase vs. HAR-1:AAPH	3.93	0.9862	ns
VOLBELLA®:Xanthine oxidase vs. HAR-1:Xanthine oxidase	−7.70	0.3162	ns
VOLBELLA®:Xanthine oxidase vs. HAR-1:Hyaluronidases	23.33	< 0.0001	****
VOLBELLA®:Xanthine oxidase vs. HAR-3:H_2O_2	0.26	> 0.9999	ns
VOLBELLA®:Xanthine oxidase vs. HAR-3:AAPH	−3.67	0.9928	ns

VOLBELLA®:Xanthine oxidase vs. HAR-3:Xanthine oxidase	–3.77	0.9908	ns
VOLBELLA®:Xanthine oxidase vs. HAR-3:Hyaluronidases	15.64	< 0.0001	****
VOLBELLA®:Hyaluronidases vs. VOLUMA®:H_2O_2	–39.19	< 0.0001	****
VOLBELLA®:Hyaluronidases vs. VOLUMA®:AAPH	–27.77	< 0.0001	****
VOLBELLA®:Hyaluronidases vs. VOLUMA®:Xanthine oxidase	–8.32	0.2065	ns
VOLBELLA®:Hyaluronidases vs. VOLUMA®:Hyaluronidases	–14.33	0.0005	***
VOLBELLA®:Hyaluronidases vs. HAR-1:H_2O_2	–41.03	< 0.0001	****
VOLBELLA®:Hyaluronidases vs. HAR-1:AAPH	–34.70	< 0.0001	****
VOLBELLA®:Hyaluronidases vs. HAR-1:Xanthine oxidase	–46.32	< 0.0001	****
VOLBELLA®:Hyaluronidases vs. HAR-1:Hyaluronidases	–15.29	0.0001	***
VOLBELLA®:Hyaluronidases vs. HAR-3:H_2O_2	–38.37	< 0.0001	****
VOLBELLA®:Hyaluronidases vs. HAR-3:AAPH	–42.30	< 0.0001	****
VOLBELLA®:Hyaluronidases vs. HAR-3:Xanthine oxidase	–42.39	< 0.0001	****
VOLBELLA®:Hyaluronidases vs. HAR-3:Hyaluronidases	–22.98	< 0.0001	****
VOLUMA®:H_2O_2 vs. VOLUMA®:AAPH	11.42	0.0122	*
VOLUMA®:H_2O_2 vs. VOLUMA®:Xanthine oxidase	30.87	< 0.0001	****
VOLUMA®:H_2O_2 vs. VOLUMA®:Hyaluronidases	24.86	< 0.0001	****
VOLUMA®:H_2O_2 vs. HAR-1:H_2O_2	–1.84	> 0.9999	ns
VOLUMA®:H_2O_2 vs. HAR-1:AAPH	4.49	0.9560	ns
VOLUMA®:H_2O_2 vs. HAR-1:Xanthine oxidase	–7.13	0.4400	ns
VOLUMA®:H_2O_2 vs. HAR-1:Hyaluronidases	23.90	< 0.0001	****
VOLUMA®:H_2O_2 vs. HAR-3:H_2O_2	0.82	> 0.9999	ns
VOLUMA®:H_2O_2 vs. HAR-3:AAPH	–3.11	0.9988	ns
VOLUMA®:H_2O_2 vs. HAR-3:Xanthine oxidase	–3.20	0.9983	ns
VOLUMA®:H_2O_2 vs. HAR-3:Hyaluronidases	16.21	< 0.0001	****
VOLUMA®:AAPH vs. VOLUMA®:Xanthine oxidase	19.45	< 0.0001	****
VOLUMA®:AAPH vs. VOLUMA®:Hyaluronidases	13.44	0.0013	**
VOLUMA®:AAPH vs. HAR-1:H_2O_2	–13.26	0.0016	**
VOLUMA®:AAPH vs. HAR-1:AAPH	–6.93	0.4888	ns
VOLUMA®:AAPH vs. HAR-1:Xanthine oxidase	–18.55	< 0.0001	****
VOLUMA®:AAPH vs. HAR-1:Hyaluronidases	12.48	0.0039	**
VOLUMA®:AAPH vs. HAR-3:H_2O_2	–10.60	0.0284	*
VOLUMA®:AAPH vs. HAR-3:AAPH	–14.53	0.0004	***
VOLUMA®:AAPH vs. HAR-3:Xanthine oxidase	–14.62	0.0003	***
VOLUMA®:AAPH vs. HAR-3:Hyaluronidases	4.78	0.9285	ns
VOLUMA®:Xanthine oxidase vs. VOLUMA®:Hyaluronidases	–6.01	0.7132	ns
VOLUMA®:Xanthine oxidase vs. HAR-1:H_2O_2	–32.71	< 0.0001	****
VOLUMA®:Xanthine oxidase vs. HAR-1:AAPH	–26.38	< 0.0001	****
VOLUMA®:Xanthine oxidase vs. HAR-1:Xanthine oxidase	–38.00	< 0.0001	****
VOLUMA®:Xanthine oxidase vs. HAR-1:Hyaluronidases	–6.97	0.4773	ns
VOLUMA®:Xanthine oxidase vs. HAR-3:H_2O_2	–30.05	< 0.0001	****
VOLUMA®:Xanthine oxidase vs. HAR-3:AAPH	–33.98	< 0.0001	****

VOLUMA®:Xanthine oxidase vs. HAR-3:Xanthine oxidase	–34.07	< 0.0001	****
VOLUMA®:Xanthine oxidase vs. HAR-3:Hyaluronidases	–14.66	0.0003	***
VOLUMA®:Hyaluronidases vs. HAR-1:H_2O_2	–26.70	< 0.0001	****
VOLUMA®:Hyaluronidases vs. HAR-1:AAPH	–20.37	< 0.0001	****
VOLUMA®:Hyaluronidases vs. HAR-1:Xanthine oxidase	–31.99	< 0.0001	****
VOLUMA®:Hyaluronidases vs. HAR-1:Hyaluronidases	–0.96	> 0.9999	ns
VOLUMA®:Hyaluronidases vs. HAR-3:H_2O_2	–24.04	< 0.0001	****
VOLUMA®:Hyaluronidases vs. HAR-3:AAPH	–27.97	< 0.0001	****
VOLUMA®:Hyaluronidases vs. HAR-3:Xanthine oxidase	–28.06	< 0.0001	****
VOLUMA®:Hyaluronidases vs. HAR-3:Hyaluronidases	–8.65	0.1610	ns
HAR-1:H_2O_2 vs. HAR-1:AAPH	6.33	0.6355	ns
HAR-1:H_2O_2 vs. HAR-1:Xanthine oxidase	–5.29	0.8587	ns
HAR-1:H_2O_2 vs. HAR-1:Hyaluronidases	25.73	< 0.0001	****
HAR-1:H_2O_2 vs. HAR-3:H_2O_2	2.66	0.9998	ns
HAR-1:H_2O_2 vs. HAR-3:AAPH	–1.27	> 0.9999	ns
HAR-1:H_2O_2 vs. HAR-3:Xanthine oxidase	–1.36	> 0.9999	ns
HAR-1:H_2O_2 vs. HAR-3:Hyaluronidases	18.05	< 0.0001	****
HAR-1:AAPH vs. HAR-1:Xanthine oxidase	–11.62	0.0099	**
HAR-1:AAPH vs. HAR-1:Hyaluronidases	19.40	< 0.0001	****
HAR-1:AAPH vs. HAR-3:H_2O_2	–3.68	0.9928	ns
HAR-1:AAPH vs. HAR-3:AAPH	–7.60	0.3353	ns
HAR-1:AAPH vs. HAR-3:Xanthine oxidase	–7.69	0.3160	ns
HAR-1:AAPH vs. HAR-3:Hyaluronidases	11.71	0.0090	**
HAR-1:Xanthine oxidase vs. HAR-1:Hyaluronidases	31.03	< 0.0001	****
HAR-1:Xanthine oxidase vs. HAR-3:H_2O_2	7.95	0.2677	ns
HAR-1:Xanthine oxidase vs. HAR-3:AAPH	4.02	0.9829	ns
HAR-1:Xanthine oxidase vs. HAR-3:Xanthine oxidase	3.93	0.9863	ns
HAR-1:Xanthine oxidase vs. HAR-3:Hyaluronidases	23.34	< 0.0001	****
HAR-1:Hyaluronidases vs. HAR-3:H_2O_2	–23.08	< 0.0001	****
HAR-1:Hyaluronidases vs. HAR-3:AAPH	–27.00	< 0.0001	****
HAR-1:Hyaluronidases vs. HAR-3:Xanthine oxidase	–27.10	< 0.0001	****
HAR-1:Hyaluronidases vs. HAR-3:Hyaluronidases	–7.69	0.3179	ns
HAR-3:H_2O_2 vs. HAR-3:AAPH	–3.93	0.9864	ns
HAR-3:H_2O_2 vs. HAR-3:Xanthine oxidase	–4.02	0.9830	ns
HAR-3:H_2O_2 vs. HAR-3:Hyaluronidases	15.39	0.0001	***
HAR-3:AAPH vs. HAR-3:Xanthine oxidase	–0.09	> 0.9999	ns
HAR-3:AAPH vs. HAR-3:Hyaluronidases	19.32	< 0.0001	****
HAR-3:Xanthine oxidase vs. HAR-3:Hyaluronidases	19.41	< 0.0001	****

[1] A significance level described by one asterisk "*" corresponded to a *p*-value between 0.01 and 0.05. A significance level described by two asterisks "**" corresponded to a *p*-value between 0.001 and 0.01. A significance level described by three asterisks "***" corresponded to a *p*-value between 0.0001 and 0.001. A significance level described by four asterisks "****" corresponded to a *p*-value inferior to 0.0001.

Table S5. Quantitative results of the post hoc Tukey's multiple comparison test, in relation with the comparative cohesivity data (i.e., drop-weight assay) presented in Figure 3A and 3B. Non-significant differences corresponded to a *p*-value > 0.05. G, gauge; ns, non-significant; Pa, Pascals.

Parameters	Compared Groups	Mean Absolute Difference (mg)	Adjusted *p*-Value	Significance Level [1]
30 G Needle 12 mm·min^{-1} Extrusion	VOLUMA® vs. HAR-1	−5.124	< 0.0001	****
	VOLUMA® vs. HAR-3	−7.716	< 0.0001	****
	VOLUMA® vs. VOLBELLA®	−5.022	< 0.0001	****
	HAR-1 vs. HAR-3	−2.592	0.0026	**
	HAR-1 vs. VOLBELLA®	0.103	0.9961	ns
	HAR-3 vs. VOLBELLA®	2.694	0.0021	**
18 G Needle 7.5 mm·min^{-1} Extrusion	VOLUMA® vs. HAR-1	0.267	0.9341	ns
	VOLUMA® vs. HAR-3	−13.700	< 0.0001	****
	VOLUMA® vs. VOLBELLA®	3.333	0.0004	***
	HAR-1 vs. HAR-3	−13.970	< 0.0001	****
	HAR-1 vs. VOLBELLA®	3.067	0.0007	***
	HAR-3 vs. VOLBELLA®	17.030	< 0.0001	****

[1] A significance level described by two asterisks "**" corresponded to a *p*-value between 0.001 and 0.01. A significance level described by three asterisks "***" corresponded to a *p*-value between 0.0001 and 0.001. A significance level described by four asterisks "****" corresponded to a *p*-value inferior to 0.0001.

Table S6. Quantitative results of the post hoc Tukey's multiple comparison test, in relation with the comparative cellular assay data (i.e., WST-1 cellular viability assays) presented in Figure 4A. Non-significant differences corresponded to a *p*-value > 0.05. ns, non-significant; Pa, Pascals; PBS, phosphate-buffered saline.

Compared Groups	Mean Absolute Difference (%)	Adjusted *p*-Value	Significance Level [1]
PBS vs. HAR-1	–32.07	0.4014	ns
PBS vs. HAR-3	–45.83	0.1207	ns
PBS vs. Radiesse®	–140.30	< 0.0001	****
PBS vs. Sculptra™	–75.34	0.0065	**
PBS vs. VOLUMA®	–51.86	0.1144	ns
HAR-1 vs. HAR-3	–13.75	0.9496	ns
HAR-1 vs. Radiesse®	–108.30	0.0003	***
HAR-1 vs. Sculptra™	–43.27	0.1537	ns
HAR-1 vs. VOLUMA®	–19.79	0.8710	ns
HAR-3 vs. Radiesse®	–94.52	0.0011	**
HAR-3 vs. Sculptra™	–29.52	0.4828	ns
HAR-3 vs. VOLUMA®	–6.04	0.9993	ns
Radiesse® vs. Sculptra™	65.00	0.0181	*
Radiesse® vs. VOLUMA®	88.48	0.0046	**
Sculptra™ vs. VOLUMA®	23.48	0.7759	ns

[1] A significance level described by one asterisk "*" corresponded to a *p*-value between 0.01 and 0.05. A significance level described by two asterisks "**" corresponded to a *p*-value between 0.001 and 0.01. A significance level described by three asterisks "***" corresponded to a *p*-value between 0.0001 and 0.001. A significance level described by four asterisks "****" corresponded to a *p*-value inferior to 0.0001.

Table S7. Quantitative results of the post hoc Tukey's multiple comparison test, in relation with the comparative cellular assay data (i.e., total collagen contents) presented in Figure 4B. Non-significant differences corresponded to a *p*-value > 0.05. ns, non-significant; Pa, Pascals; PBS, phosphate-buffered saline.

**Compared Groups**	**Mean Absolute Difference** (µg/mL)	**Adjusted *p*-Value**	**Significance Level** [1]
PBS vs. HAR-1	–24.06	0.0044	**
PBS vs. HAR-3	–19.64	0.0023	**
PBS vs. Radiesse®	–4.23	0.9508	ns
PBS vs. Sculptra™	–10.16	0.3508	ns
PBS vs. VOLUMA®	–3.05	0.9878	ns
HAR-1 vs. HAR-3	4.43	0.9519	ns
HAR-1 vs. Radiesse®	19.84	0.0346	*
HAR-1 vs. Sculptra™	13.91	0.2157	ns
HAR-1 vs. VOLUMA®	21.01	0.0233	*
HAR-3 vs. Radiesse®	15.41	0.0433	*
HAR-3 vs. Sculptra™	9.48	0.3696	ns
HAR-3 vs. VOLUMA®	16.59	0.0267	*
Radiesse® vs. Sculptra™	–5.93	0.8723	ns
Radiesse® vs. VOLUMA®	1.18	> 0.9999	ns
Sculptra™ vs. VOLUMA®	7.11	0.7682	ns

[1] A significance level described by one asterisk "*" corresponded to a *p*-value between 0.01 and 0.05. A significance level described by two asterisks "**" corresponded to a *p*-value between 0.001 and 0.01.

Table S8. Quantitative results of the post hoc Tukey's multiple comparison test, in relation with the comparative cellular assay data (i.e., total protein contents) presented in Figure 4C. Non-significant differences corresponded to a p-value > 0.05. ns, non-significant; Pa, Pascals; PBS, phosphate-buffered saline.

**Compared Groups**	**Mean Absolute Difference** (mg/mL)	**Adjusted *p*-Value**	**Significance Level** [1]
PBS vs. HAR-1	–1.01	0.4439	ns
PBS vs. HAR-3	–1.33	0.2006	ns
PBS vs. Radiesse®	–1.24	0.2557	ns
PBS vs. Sculptra™	0.11	> 0.9999	ns
PBS vs. VOLUMA®	0.57	0.8784	ns
HAR-1 vs. HAR-3	–0.32	0.9871	ns
HAR-1 vs. Radiesse®	–0.23	0.9972	ns
HAR-1 vs. Sculptra™	1.11	0.3504	ns
HAR-1 vs. VOLUMA®	1.58	0.1023	ns
HAR-3 vs. Radiesse®	0.09	> 0.9999	ns
HAR-3 vs. Sculptra™	1.44	0.1510	ns
HAR-3 vs. VOLUMA®	1.90	0.0401	*
Radiesse® vs. Sculptra™	1.34	0.1945	ns
Radiesse® vs. VOLUMA®	1.81	0.0525	ns
Sculptra™ vs. VOLUMA®	0.46	0.9425	ns

[1] A significance level described by one asterisk "*" corresponded to a p-value between 0.01 and 0.05.
